# A novel immune score model predicting the prognosis and immunotherapy response of breast cancer

**DOI:** 10.1038/s41598-023-31153-2

**Published:** 2023-04-19

**Authors:** Wenchang Lv, Xiao He, Yichen Wang, Chongru Zhao, Menglu Dong, Yiping Wu, Qi Zhang

**Affiliations:** 1 Department of Plastic Surgery, Tongji Hospital, Tongji Medical College, Huazhong University of Science and Technology, 1095 Jiefang Avenue, Wuhan, 430030 Hubei China; 2 Department of Thyroid and Breast Surgery, Tongji Hospital, Tongji Medical College, Huazhong University of Science and Technology, 1095 Jiefang Avenue, Wuhan, 430030 Hubei China

**Keywords:** Cancer, Cell biology, Computational biology and bioinformatics, Genetics, Immunology, Biomarkers

## Abstract

Breast cancer (BC) is one of the most common malignancies. However, the existing pathological grading system cannot accurately and effectively predict the survival rate and immune checkpoint treatment response of BC patients. In this study, based on The Cancer Genome Atlas (TCGA) database, a total of 7 immune-related genes (IRGs) were screened out to construct a prognostic model. Subsequently, the clinical prognosis, pathological characteristics, cancer-immunity cycle, tumour immune dysfunction and exclusion (TIDE) score, and immune checkpoint inhibitor (ICI) response were compared between the high- and low-risk groups. In addition, we determined the potential regulatory effect of NPR3 on BC cell proliferation, migration, and apoptosis. The model consisting of 7 IRGs was an independent prognostic factor. Patients with lower risk scores exhibited longer survival times. Moreover, the expression of NPR3 was increased but the expression of PD-1, PD-L1, and CTLA-4 was decreased in the high-risk group compared to the low-risk group. In addition, compared with si-NC, si-NPR3 suppressed proliferation and migration but promoted apoptosis in both MDA-MB-231 and MCF-7 cells. This study presents a model for predicting survival outcomes and provides a strategy to guide effective personalized immunotherapy in BC patients.

## Introduction

Breast cancer (BC) is one of the most common malignancies and is a dominant cause of cancer-related mortality around the world^1^. Therapies for combating BC involve multidisciplinary approaches, such as surgery, radiotherapy, chemotherapy, endocrine therapy, and immunotherapy^2^. These approaches have greatly reduced the mortality rate of BC patients, but the prognosis of advanced BC patients is still unsatisfactory due to tumour relapse, metastasis, and drug resistance. The existing tumour-node-metastasis (TNM) staging system is the gold standard for BC diagnosis, but to some extent, it does not provide accurate prognostic information. Therefore, effective monitoring and prediction tools for BC prognosis are urgently needed for the BC classification and treatment guidance.

BC is a highly heterogeneous entity with unique tumour microenvironment (TME) characteristics. The BC TME is characterized by spatial heterogeneity and includes multiple secreted factors and cell types, including tumour cells, vascular cells, stromal cells, and immune cells^3^. The TME is greatly associated with proliferation, angiogenesis, immune system inhibition, and the response to therapy^4^. The immunosuppressive TME enables and preconditions the tumour cell seed to propagate lymphatically and hematologically, resulting in distant metastasis and an undesirable prognosis^5^. Elucidating the TME immune infiltration features TME could reveal the role of immune cells and immune infiltration characteristics and help to establish a good model for predicting BC prognosis.

Cancer prognosis is closely related to immune-related genes (IRGs). Sun et al. identified IRGs of papillary thyroid cancer (PTC) that could predict progression-free survival (PFS) and provide information on altered signal pathways, mutational patterns, and potential drugs for PTC^6^. Currently, many novel immunotherapies based on immune checkpoint inhibitors (ICIs) are available for BC treatment; these include PD-1, PD-L1, and CTLA-4 inhibitors^7^. The expression level and activity of these immune checkpoints in the TME profoundly affect tumour progression and the response to ICI treatment. For example, as there is a large difference in the proportion of PD-L1-positive cells between primary BC tissue and metastatic BC tissue, the assessment of PD-L1 expression can help judge treatment efficacy and prognosis when selecting patients for immunotherapy^8^. Jin et al. established a prognostic risk model based on 12 IRGs, showing the potential to stratify patients into different risk groups^9^. Therefore, IRGs have important clinical implications as prognostic biomarkers for BC patients^10^. Additionally, a deeper understanding of IRGs in the BC TME is vital for exploiting therapeutic interventions to improve the prognosis of BC patients.

At present, some studies have explored IRGs and their relationship with immune cell infiltration, the TME, and prognosis in BC cohorts. To address this issue, in the present study, a prognostic model based on 7 IRGs (ULBP2, CCL24, TSLP, FLT3, NPR3, TNFRSF8, and ANO6) was established through least absolute shrinkage and selection operator (LASSO) penalized Cox regression analysis. The time-dependent receiver operating characteristic (ROC) curve revealed that the IRG prognostic model demonstrated better predictive performance in terms of overall survival (OS) than other independent clinicopathological variables, including age, clinical stage, and TNM stage. Then, those BC patients from The Cancer Genome Atlas (TCGA) and IMvigor210 cohorts were classified into low- and high-risk groups based on the median risk scores of the IRG prognostic model. Furthermore, we explored the differences in clinical outcomes, genetic variants, biological processes, immune cell infiltration, TME, immunotherapy effect, and drug sensitivity between the high- and low-risk subgroups. Overall, prognosis evaluation based on the IRG model allows the consideration of BC TME features when predicting clinical outcomes.

## Materials and methods

### Data collection and gene selection

A total of 1097 BC RNA sequencing (RNA-Seq) expression profile datasets and the corresponding clinicopathological characteristics were downloaded from the TCGA data portal (https://tcga-data.nci.nih.gov/tcga/). The significant clinical characteristics, such as age, stage, OS, progression-free interval (PFI), and disease-specific survival (DSS) of BC are summarized and displayed in Table S1. Moreover, the gene expression matrix and survival time were acquired from a website (http://research-pub.gene. com/IMvigor210CoreBiologies/) were used for the testing cohort. Subsequently, a gene set of 6196 IRGs was extracted from the ImmPort database (http://www.immport.org/), which is a the professional database of immune regulators and markers. Detailed information on IRGs is shown in Table S2. Moreover, the limma R package was used to identify differentially expressed IRGs between tumour and normal tissues (|log2 FC|> 1 and FDR < 0.05).

### Functional enrichment analysis

We performed Gene Ontology (GO) and Kyoto Encyclopedia of Genes and Genomes (KEGG) enrichment analyses to identify the cellular components, biological processes, molecular functions, and signalling pathways using the R package “clusterprofiler”^11^. The source of the KEGG was www.kegg.jp/kegg/kegg1.html. We got permission from the website: www.kegg.jp/feedback/copyright.html^12^. Moreover, based on the annotation files “c5.go.v7.4.symbols.gmt” and “c2.cp.kegg.v7.4.symbols.gmt”, Gene Set Enrichment Analysis (GSEA) was conducted to verify potential biological processes between the high-risk group and low-risk group using the R package “enrichplot”. Moreover, principal component analysis (PCA) with the R package “scatterplot3d” was performed on these samples together with our genotyped samples. A false discovery P < 0.05 was set as the cut-off.

### Construction and validation of a prognostic IRG model

First, the univariate Cox analysis of OS was applied to verify the potential IRGs by R “survival” filtered based on P  < 0.05. Second, we employed LASSO logistic regression to filter out hub genes to improve the stability of the risk score model by the R package “glmnet”. Third, a prognostic IRG model was constructed based on coefficients (β) calculated from the multivariate Cox regression as the weights. Briefly, the risk score for each BC patient in the TCGA cohort was calculated using the risk formula: risk score = expression of gene*1* × β1 + expression of gene*2* × β2 + ⋯ expression of gene*n* × βn. Then, we divided the patients into high- and low-risk groups according to the median risk score. Finally, the ROC curve was generated, and the area under the curve (AUC) were calculated to evaluate the prediction Cox model for the prognostic biomarkers in BC patients.

### Evaluation of immune infiltration level

The CIBERSORT deconvolution algorithm (https://cibersort.stanford.edu/) is a widely accepted computing method to analyse immunological characteristics based on hundreds of immunomarkers. In this study, a gene model matrix with interpretation, known as LM22, was used to annotate the proportion of tumour-infiltrating lymphocytes between the high- and low-risk subgroups. Based on the annotation files “commonGenes.gct” and “estimateScore.gct”, the tumour purity, ESTIMATE score, immune score, and stromal score were evaluated using the R package “estimate”.

### Immunotherapy/chemotherapy response prediction

As reported in previous articles, the tumour immune dysfunction and exclusion (TIDE) algorithm (http://tide.dfci.harvard.edu/) was used to predict the immune therapy response between the high- and low-risk groups in the TCGA database. Patients with higher TIDE scores had a higher chance of antitumour immune escape, thereby indicating a lower immunotherapy response rate. Based on the public drug sensitivity databases, we also predicted the chemotherapy response of each sample based on Genomics of Drug Sensitivity in Cancer (GDSC) (https://www.cancerrxgene.org/) by the R package “Prophetic”. The prediction accuracy was estimated according to the area under the dose–response curve value of each training set. Low AUC values indicate increased sensitivity to chemotherapeutic treatment.

### Cell culture and transfection

In this study, we obtained the human breast carcinoma cell lines MCF-7 and MDA-MB-231 from the American Type Culture Collection (ATCC). According to previous research, MCF-7 and MDA-MB-231 cells were cultured in Dulbecco’s modified Eagle medium (DMEM) (Gibco, Grand Island, NY) supplemented with 10% foetal bovine serum (FBS) at 37 °C. The synthesized small interfering RNA (siRNA) was dissolved in solution at a concentration of 20 μmol/L. MCF-7 and MDA-MB-231 cells were seeded in 6-well plates at a density of 4 × 10^5^/mL for 24 h. Before transfection, the tumour cells were rinsed with serum-free and antibiotic-free DMEM. According to the reagent instructions, siRNA was transfected into cells by Lipofectamine 3000 (Invitrogen, Carlsbad, CA, USA). After 24 h of transfection, the silencing efficiency was tested in subsequent experiments. All siRNA sequences for NPR3 are available in Table S3.

### RNA extraction and RT‒PCR

First, we extracted total RNA from BC cell lines and tissues using TRIzol Reagent (Invitrogen). Second, isolated RNA was reverse transcribed into cDNA using High Capacity cDNA Reverse Transcription kits (Vazyme, China) according to the manufacturer’s recommendations. Third, quantitative reverse transcription polymerase chain reaction (qRT‒PCR) was carried out with SYBR Green PCR Master Mix (Vazyme, China). GAPDH was used as an internal control. All the primers of IRGs in the risk model are displayed in Table S4.

### Proliferation and migration assay

After siRNA transfection, BC cells of different groups were inoculated into 96-well plates (3 × 10^4^ cells per well). The cells were cultured with serum-free medium in an incubator, and 10 μL of Cell Counting Kit-8 (CCK-8) reagent was added after incubation for 1 day, 2 day, and 3 day. Then, the cells were incubated in the dark for 1–4 h. The absorbance at 450 nm was measured using an automatic microplate reader (Synergy4; BioTek, USA). Moreover, BC cell lines were seeded into a 6-well plate and then scratched with a 1 mL pipette tip when cell fusion approached 90%. After treated with 1 μg/mL mitomycin C in serum-free medium for 24 h, the width of the wounds was examined under a microscope (ICX41, SDPTOP, China) (magnification, 40×)^13^. The wounds were photographed at 0 h and 24 h after injury. Then, the wound widths and wound closure rates were measured by ImageJ software. The relative closure rate was calculated using (W_0_ − W_t_)/W_0_ × 100%, where W_0_ represented the initial wound width and W_t_ represented the wound width after incubation with serum-free medium for 24 h. Transwell migration assay was also performed to assess cellular migration ability using a 6.5-mm diameter Transwell chamber (Corning, USA). MCF-7 and MDA-MB-231 cells (5 × 10^5^) in 200 μL serum-free DMEM were seeded into the upper chamber in the Transwell chamber, and 700 μL DMEM with 10% FBS was added into the lower chamber. After incubating at 37 °C for 24 h, the membranes of the chamber were fixed with 4% paraformaldehyde for 30 min, and the upper side of the chamber was wiped with cotton swabs. Then, 0.1% crystal violet was used to stain the migrated cells on the lower side of the membrane for 30 min at 37 °C. Subsequently, an inverted biological microscope (ICX41, SDPTOP, China) was used to count the cell number (magnification, 200×). Five areas were randomly selected in each group. All experiments were performed independently three times. Data are shown as the mean ± standard deviation (SD).

### Detection of apoptosis

Cell apoptosis was detected by an Annexin V-fluorescein isothiocyanate (FITC)/propidium iodide (PI) apoptosis kit (MultiSciences, China) with flow cytometry. Cells were seeded in 6-well plates and transfected with siRNAs at the indicated concentrations for 24 h. Then approximately 1 × 10^5^ cells, including suspended cells, were harvested by ethylene diamine-tetra acetic acid (EDTA)-free trypsinization and washed twice with cold phosphate buffer saline (PBS). After resuspension in 500 μL binding buffer, cells were stained with 5 μL Annexin-V-FITC and 5 μL PI in dark conditions at room temperature for 15 min. Finally, cell apoptosis was detected by a CytoFLEX-3 flow cytometer (BD, USA). Acquired data were analysed using FlowJo software. Three replicates were set in each group, and all experiments were performed independently 3 times.

### Immunohistochemistry and immunofluorescence of tissue samples

We collected BC tissue samples and clinical data from the high- and low-risk groups. For immunohistochemistry (IHC), human mammary tissues were deparaffinized in xylene, rehydrated in different concentrations of ethanol solutions and heated in citrate buffer using a pressure cooker for antigen retrieval. Next, the sections were immunostained with primary antibodies against human NPR3, PD-1, PD-L1, and CTLA-4 (1:100; Proteintech, China) overnight at 4 °C followed by horseradish peroxidase (HRP)-conjugated secondary antibodies. Peroxidase activity was visualized using the Diaminobenzidine (DAB) Peroxidase Substrate Kit (Maxin, China), and the sections were counterstained with haematoxylin. A SOPTOP CX40 microscope (China) was used to capture digital images of the sections. For immunofluorescence (IF) analysis, the procedures for managing human mammary tissues before staining were the same as those for IHC. The mammary sections were incubated overnight at 4 °C with a cocktail of primary antibodies against human NPR3 (1:100; Proteintech, China), followed by incubation with a cocktail of secondary antibodies (Life Technologies, CA, USA) for 1 h at room temperature on the next day. The sections were then incubated with human anti-CD206 (1:100; Proteintech, China) and nuclear 4,6-diamidino-2-phenylindole (DAPI; Vector Laboratories, Burlingame) for counterstaining. Digital images were captured by using an Olympus fluorescence microscope (Japan).

### Statistical analyses

This study was conducted according to the principles of the Declaration of Helsinki. This research was approved by the Medical Ethics Committee, Tongji Hospital, Tongji Medical College, Huazhong University of Science and Technology. All statistical tests involved in this study were conducted using R software (v4.0.2, https://www.r-project.org/). Student’s t test or Pearson’s correlation coefficient was performed to measure continuous variables. The immune infiltration level between the high- and low-risk subgroups was evaluated by the Wilcox test. The time-dependent AUC and C-index were calculated by the timeROC and compareC packages, respectively. In the lab-based experiments, two groups analysis was performed one-way analysis of variance with t-test by using Graphpad Prism 8.0 (GraphPad, USA). For all statistical analyses, a two-tailed P  < 0.05 was considered significant.

### Ethics approval and consent to participate

The research was carried out following the Medical Ethics Committee, Tongji Hospital, Tongji Medical College, Huazhong University of Science and Technology. All procedures performed in studies involving human participants were in accordance with the ethical standards of the institutional research committee and exempted from informed consent.

## Results

### Identification of a prognostic risk model based on IRGs

**Figure 1:**
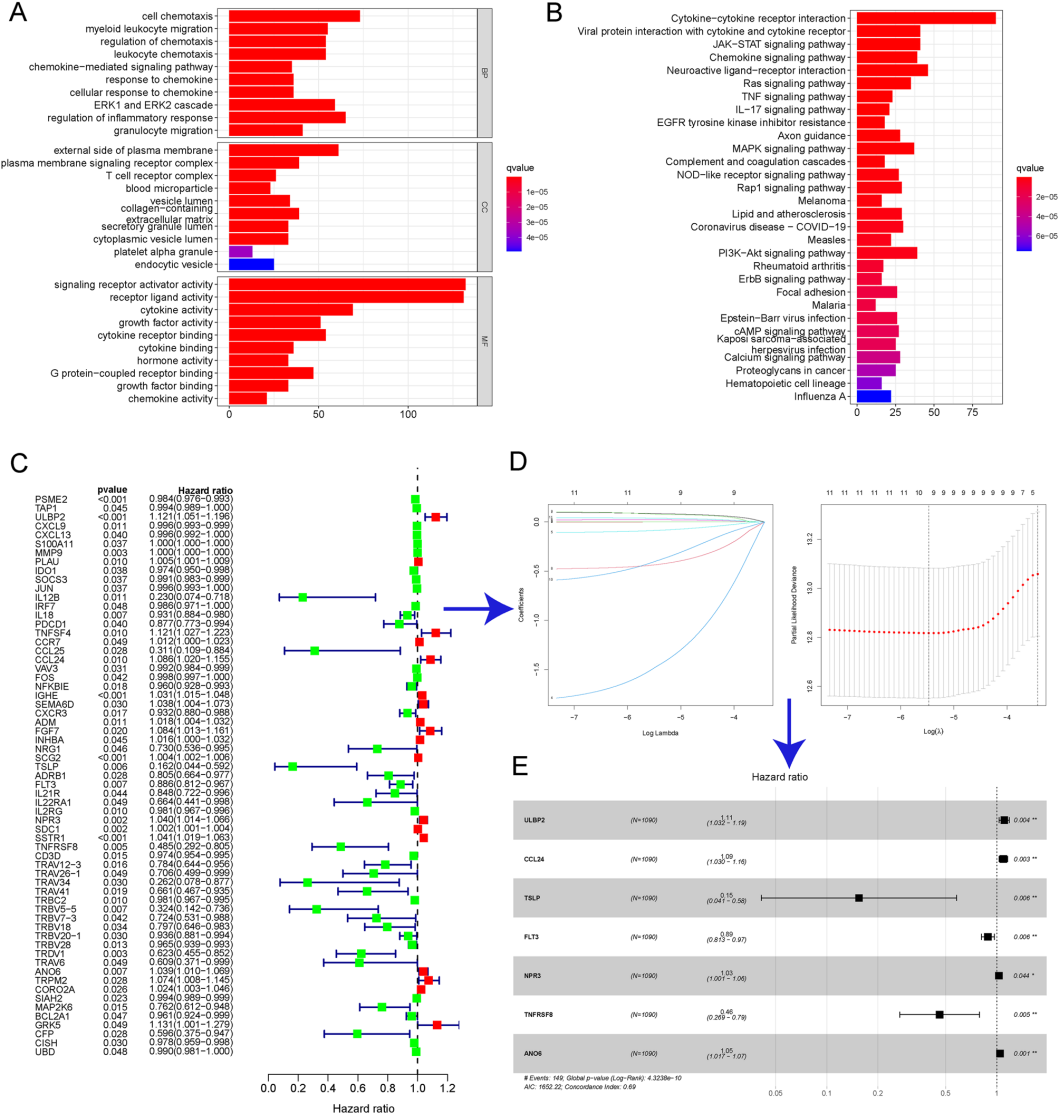
Characteristics of immune-related genes (IRGs) and construction of the IRG-related risk model in breast cancer (BC). (**A**) Gene Ontology (GO) enrichment analysis of IRGs. (**B**) Kyoto Encyclopedia of Genes and Genomes (KEGG) enrichment analysis of IRGs. The permission was obtained from the website: www.kegg.jp/feedback/copyright.html. Univariate Cox regression analysis (**C**), least absolute shrinkage and selection operator (LASSO) regression analysis (**D**), and multivariate Cox regression analysis (**E**) were applied to identify the prognostic IRGs. *P < 0.05; **P < 0.01; ***P < 0.001. *IRG* immune-related gene, *BC* breast cancer, *GO* Gene Ontology, *KEGG* Kyoto Encyclopedia of Genes and Genomes, *LASSO* least absolute shrinkage and selection operator.

First, KEGG and GO analyses were used to analyse the potential function of IRGs. The GO analysis showed that these IRGs were mainly enriched in molecular functions related to cellular interactions, such as signalling receptor activator activity and receptor-ligand activity (Fig. 1A). Moreover, these IRGs were enriched in pathways related to cytokine-cytokine receptor interactions in the KEGG analysis (Fig. 1B). Subsequently, 63 IRGs were identified as significantly correlated with BC prognosis by univariate Cox regression analysis (P  < 0.05) (Fig. 1C). After that, these 10 IRGs were subjected to LASSO regression analysis (Fig. 1D) and stepwise multivariate Cox regression analysis (Fig. 1E). Finally, a prognostic risk model was constructed based on 7 IRGs, including ULBP2, CCL24, TSLP, FLT3, NPR3, TNFRSF8, and ANO6. The coefficients of those 7 IRGs were used to calculate the risk score (Fig. 1E). The model formula was as follows: risk score = ULBP2 × (0.1011) + CCL24 × (0.0877) + TSLP × (− 1.8672) + FLT3 × (− 0.1206) + NPR3 × (0.0283) + TNFRSF8 × (− 0.7746) + ANO6 × (0.0446). Moreover, Kaplan‒Meier analysis showed that among those 7 IRGs, high expression of ULBP2, CCL24, NPR3, and ANO6 predicted a poor prognosis in BC patients, while high expression of TSLP, FLT3, and TNFRSF8 was correlated with an optimal prognosis in BC patients (Figure S1).

### Validation of the prognostic value of the immune-related risk model in BC cohort

**Figure 2:**
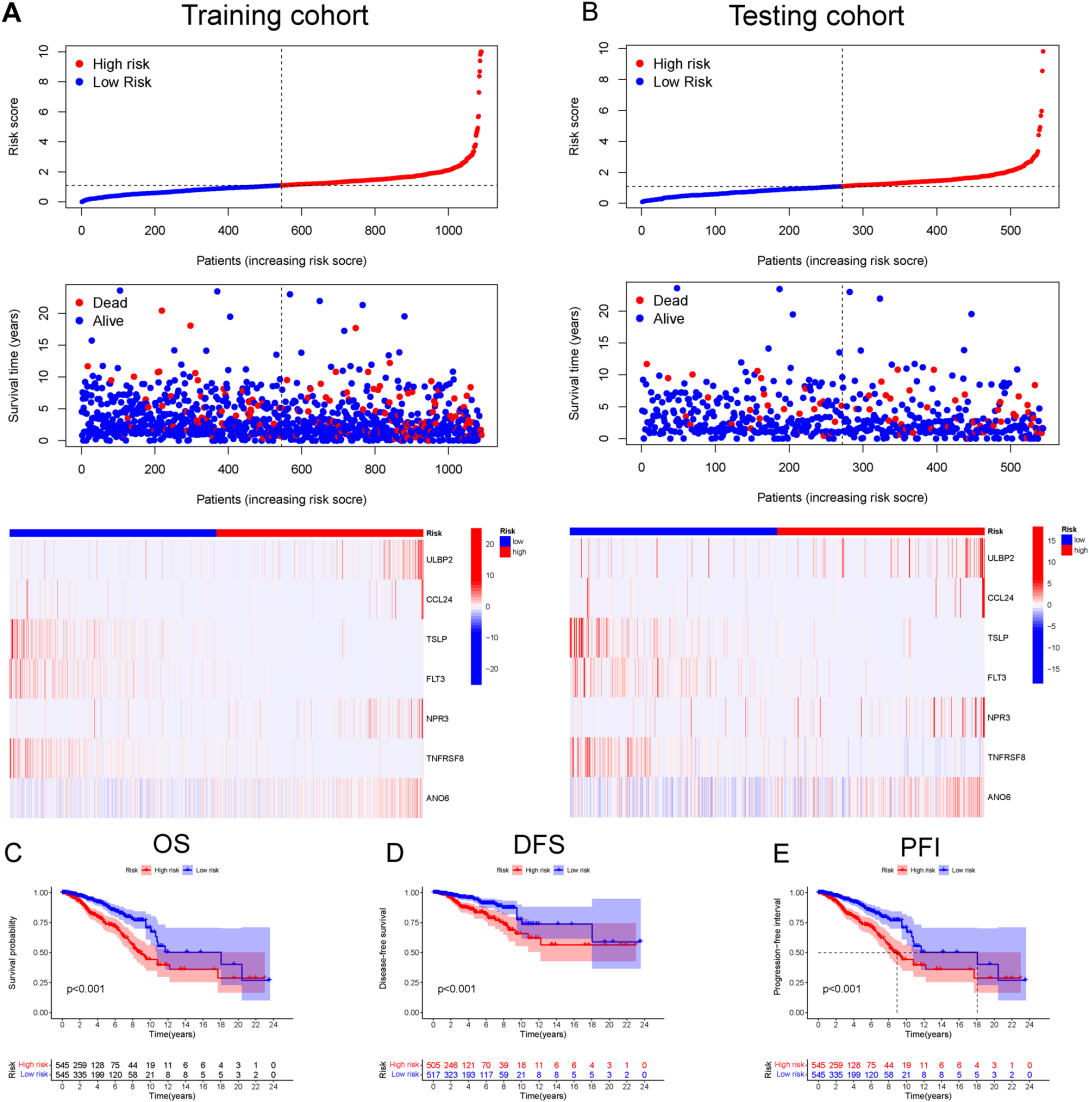
The distribution of risk score, survival status, and mRNA expression panel in the training cohort and testing cohort. In the training cohort (**A**) and testing cohort (**B**), the risk score of breast cancer (BC) patients was closely related to survival status and expression panel. Kaplan–Meier survival analysis was conducted to evaluate the differences of overall survival (OS), disease-free survival (DFS), and progression-free interval (PFI) in the high- and low-risk groups based on the “survival” and “survminer” R packages. In the training cohort, Kaplan‒Meier curves revealed the OS (**C**), DFS (**D**), and PFI (**E**) of BC patients in the high-risk group compared with those in the low-risk group. *BC* breast cancer, *OS* overall survival, *DFS* disease-free survival, *PFI* progression-free interval.

As shown in Fig. 2A, a total of 1090 BC patients were divided into high-risk (545 patients) and low-risk groups (545 patients) according to the median threshold of the risk score. Interestingly, as the risk score increased, the mortality rate in BC patients increased. Additionally, the heatmap further illustrated the differential expression pattern of these 7 IRGs between the high- and low-risk groups. Moreover, the same conclusion was reached in the testing cohort (Fig. 2B). Of note, the Kaplan‒Meier curves demonstrated that BC patients in the high-risk group had relatively shorter OS (Fig. 2C), disease-free survival (DFS) (Fig. 2D), and PFI (Fig. 2E) than patients in the low-risk group.

### Validation of the independence of the prognostic model and development of a nomogram for BC patients

**Figure 3:**
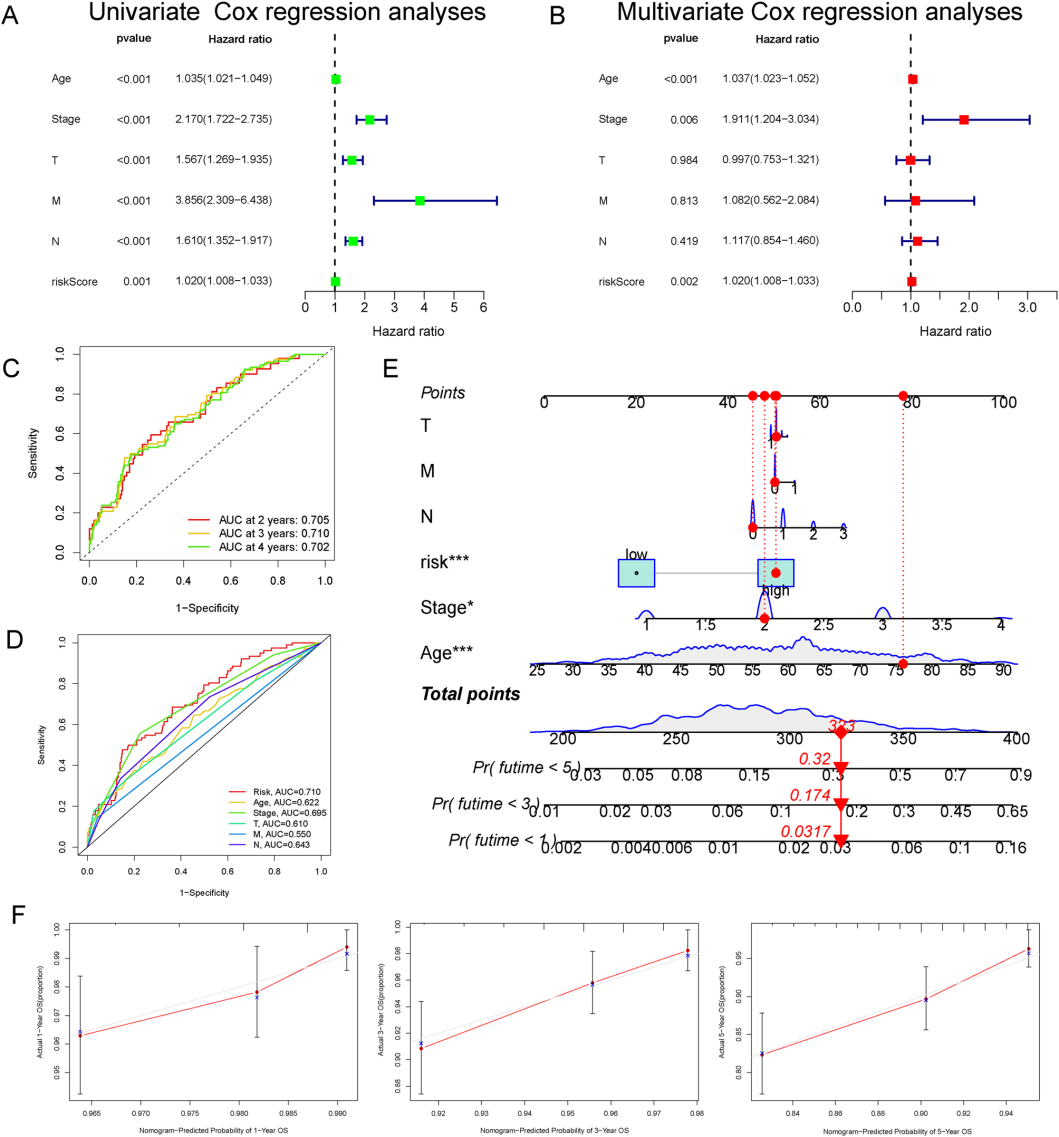
Validating the immune-related predictive model in the training cohort of breast cancer (BC) patients. Univariate (**A**) and multivariate (**B**) Cox regression analyses determined that the risk score was independently associated with poor overall survival (OS) in BC. (**C**) The receiver operating characteristic (ROC) curves of the risk model at 2-, 3- and 4-year. (**D**) The area under the curve (AUC) of the risk score for predicting that 3-year OS of BC patients was higher than that of age, clinical stage, and tumour-node-metastasis (TNM) stage. (**E**) All independent prognostic factors, including the risk score, were incorporated into the model to delineate a nomogram for predicting the 1-, 3- and 5-year individual survival rates of BC patients. (**F**) Based on the consistency between the observed and predicted values, the nomogram calibration plot of the training cohort was established. *BC* breast cancer, *OS* overall survival, *ROC* receiver operating characteristic, *AUC* area under the curve, *TNM* tumour-node-metastasis.

To verify whether the risk score could predict prognosis independent of other clinicopathological parameters, including age, clinical stage, and TNM stage, univariate and multivariate Cox regression analyses were executed. The results of the univariate Cox regression analysis revealed that the risk score was independently associated with poor OS in BC [hazard ratio (HR) = 1.020, 95% confidence interval (CI) 1.008–1.033, P  < 0.01] (Fig. 3A). The risk score also served as an independent risk factor for BC prognosis after adjusting for other confounding factors (HR = 1.020, 95% CI 1.008–1.033, P < 0.01) (Fig. 3B). In addition, the ROC curve suggested that the risk score had robust efficiency for predicting BC prognosis, with high AUC values of 0.705, 0.710, and 0.702 at 2-, 3- and 4-year, respectively (Fig. 3C). In particular, the AUC of the risk score for predicting the 3-year OS of BC patients was higher than that of the other independent clinicopathological variables, including age, clinical stage, and TNM stage (Fig. 3D). Then, all independent prognostic factors, including the risk score, were enrolled to delineate a nomogram for predicting the 1-, 3- and 5-year survival probabilities for individual BC patients (Fig. 3E). Furthermore, the calibration curve of the nomogram for predicting 1-, 3- and 5-year OS showed good consistency with actual observations, demonstrating the potential and optimal application of the nomogram in predicting the long-term survival of BC patients (Fig. 3F).

### Weighted gene co-expression network analysis (WGCNA) and identification of significant modules in BC

**Figure 4:**
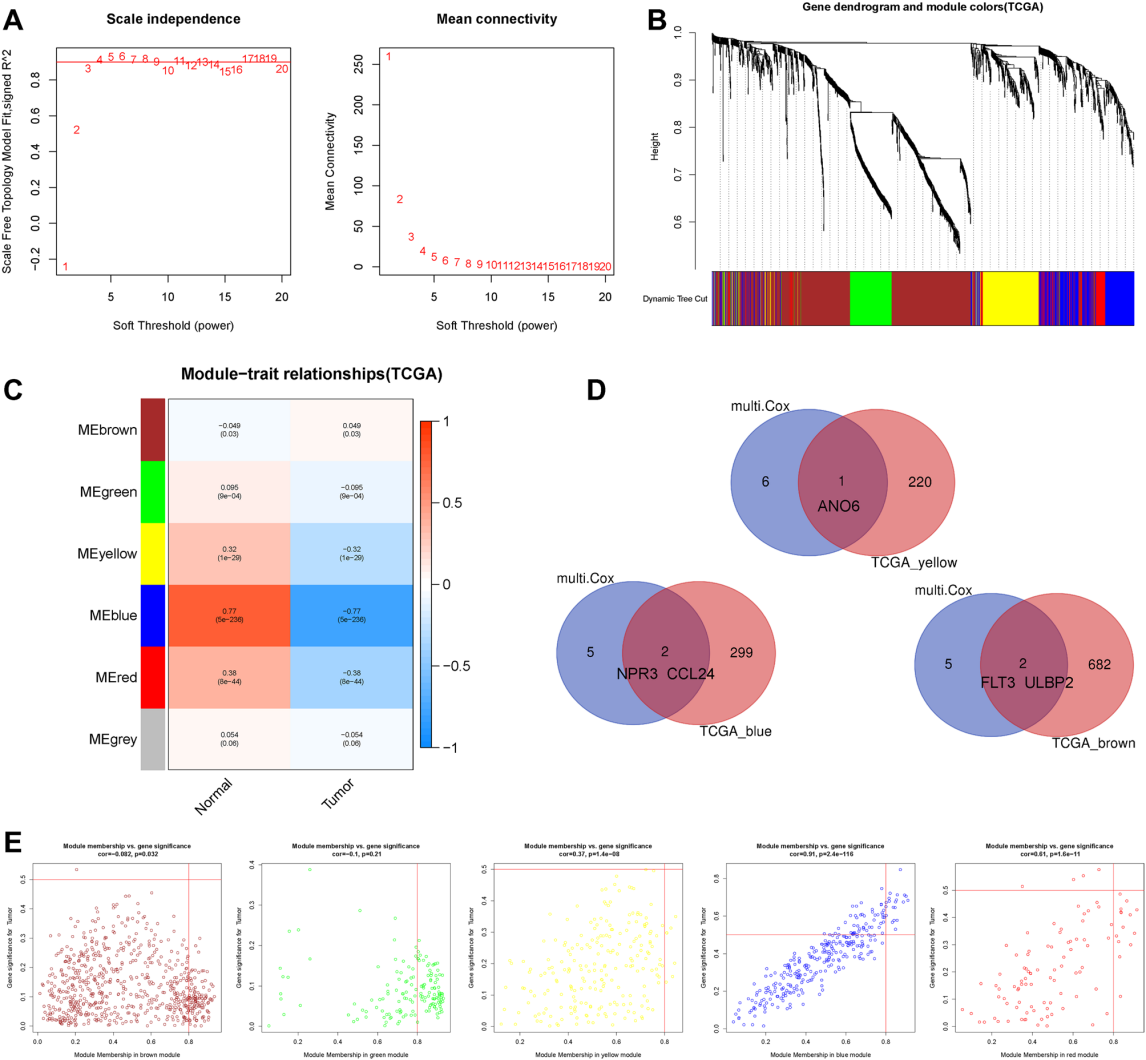
Weighted gene co-expression network analysis (WGCNA) and identification of candidate immune-related hub genes. (**A**) Analysis of network topology for various soft-thresholding powers. (**B**) Gene dendrogram and module colours. (**C**) Immune-related genes (IRGs) in the brown, green, and yellow modules were significantly correlated with BC progression. (**D**) Venn diagram results confirmed that IRGs ANO6, NPR3, CCL24, FLT3, and ULBP2 were also recruited to construct our prognostic risk model. (**E**) Module membership (MM) vs. gene significance (GS) analysis of 7 modules showed that MM was significantly correlated with GS in brown, yellow, blue, and red modules. *WGCNA* weighted gene co-expression network analysis, *IRG* immune-related gene, *MM*, module membership, *GS* gene significance.

Next, weighted gene coexpression network analysis (WGCNA) was performed to identify the candidate immune-related hub genes intimately associated with different clinical features of BC. First, the expression data of all IRGs were used to construct the gene expression similarity matrix by calculating the coefficients between gene pairs. Then, the similarity matrix was transformed into an adjacency matrix, and the soft-thresholding power was set to β = 4 (Fig. 4A). Next, the dynamic pruning tree and the recognition coexpression network were built to identify the modules based on the cut-off value = 0.25 (Fig. 4B). Finally, 7 modules were identified by setting the smallest module size as 30; the IRGs were clustered in 3 modules (brown, green, and yellow), and these modules were significantly correlated with BC features (P < 0.05) (Fig. 4C). Furthermore, those 3 modules included 5 IRGs (ANO6, NPR3, CCL24, FLT3, and ULBP2) that were also used to construct our prognostic risk model (Fig. 4D). Moreover, module membership (MM) vs. gene significance (GS) analysis of 7 modules showed that MM was significantly correlated with GS in the brown, yellow, blue, and red modules (Fig. 4E). These results emphasized the connection of the hub IRGs in our risk model with BC clinical characteristics.

### The risk score is correlated with BC clinical characteristics

**Figure 5:**
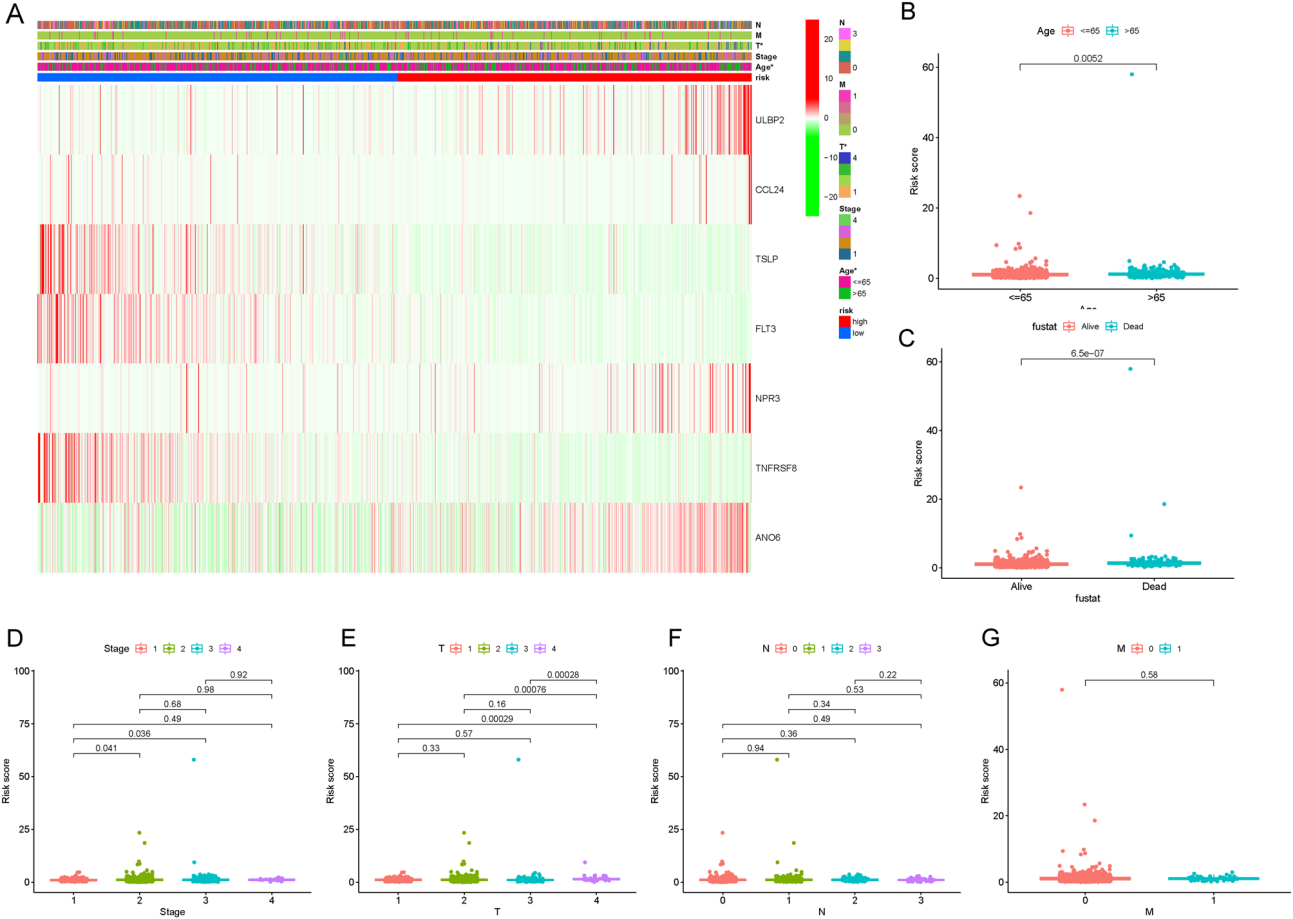
The correlation of the risk score with the clinical characteristics in the training cohort of breast cancer (BC) patients. (**A**) The heatmap displays the correlation of the 7 immune-related genes (IRGs) in our model with BC clinical characteristics. The correlation of the prognostic model with BC clinical characteristics [including tumour-node-metastasis (TNM), pathological stage, and age] between high- and low-risk groups was explored by the “limma” and “ggpubr” R packages. (**B**,**C**) The risk score was higher in BC patients aged > 65 years and those with adverse outcomes. (**D**–**G**) The risk scores of BC patients with different clinical stages and TNM stages were also significantly different. *BC* breast cancer, *IRG* immune-related gene, *TNM* tumour-node-metastasis.

As shown in Fig. 5A, the heatmap vividly displayed the correlation of the 7 IRGs in our model with BC clinical characteristics, including TNM stages, clinical stages, age and risk. In detail, the risk score was higher in BC patients aged > 65 (Fig. 5B) and those with adverse outcomes (Fig. 5C). In addition, BC patients with different clinical stages and TNM stages also had different risk scores (Fig. 5D–G). Therefore, the risk score based on our risk model might provide potential assistance in predicting clinical outcomes in BC patients.

### The high- and low-risk groups presented different biological processes

**Figure 6:**
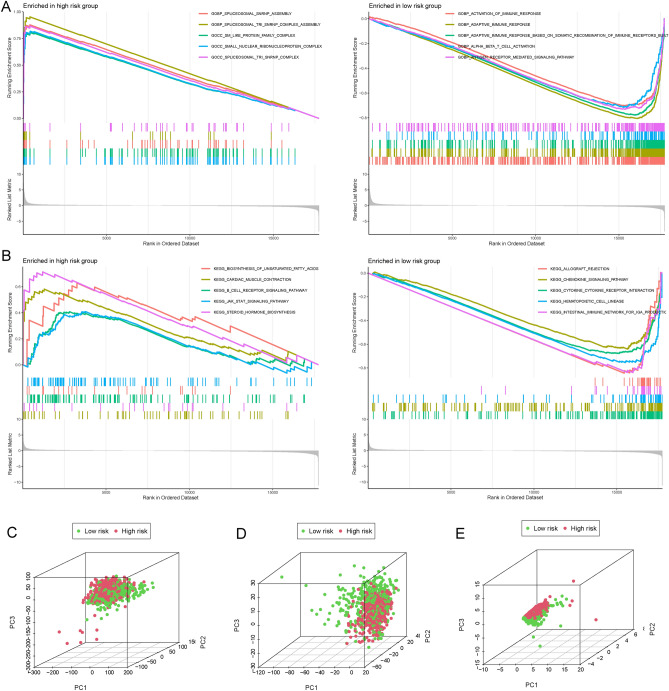
Analysis of differentially expressed genes between risk groups revealed enriched biological processes. Gene Ontology (GO) (**A**) and Kyoto Encyclopedia of Genes and Genomes (KEGG) (**B**) analyses of differentially expressed genes between the high- and low-risk groups based on the risk model in breast cancer (BC). The 3D scatterplot of principal component analysis (PCA) showed the distribution of BC patients based on the whole gene expression profile (**C**), all immune-related genes (IRGs) (**D**), and 7 IRGs (**E**). *GO* Gene Ontology, *KEGG* Kyoto Encyclopedia of Genes and Genomes, *BC* breast cancer, *PCA* principal component analysis, *IRG* immune-related gene.

GSEA was utilized to detect the potential biological function differences between the high- and low-risk subgroups. The GO analysis revealed that the genes related to a high risk score in BC patients were mainly enriched in spliceosomal snRNP assembly, spliceosomal trisnRNP complex assembly, the SM-like protein family complex, the small nuclear ribonucleoprotein complex and the spliceosomal trisnRNP complex (Fig. 6A). Additionally, in the KEGG analysis, genes related to a high risk score in BC patients were enriched in tumour progression-related processes, including biosynthesis of unsaturated fatty acids, biosynthesis of steroid hormone, cardiac muscle contraction, the B-cell receptor signalling pathway, and the JAK-STAT signalling pathways (Fig. 6B). Notably, genes related to low risk showed enrichment in the activated and adaptive immune responses (Fig. 6A,B). Furthermore, PCA showed that BC patients could be divided into two risk subgroups based on 7 IRGs (Fig. 6E) rather than the whole gene expression profile (Fig. 6C) or all IRGs (Fig. 6D).

### Genetic alternation of IRGs between the two risk groups

**Figure 7:**
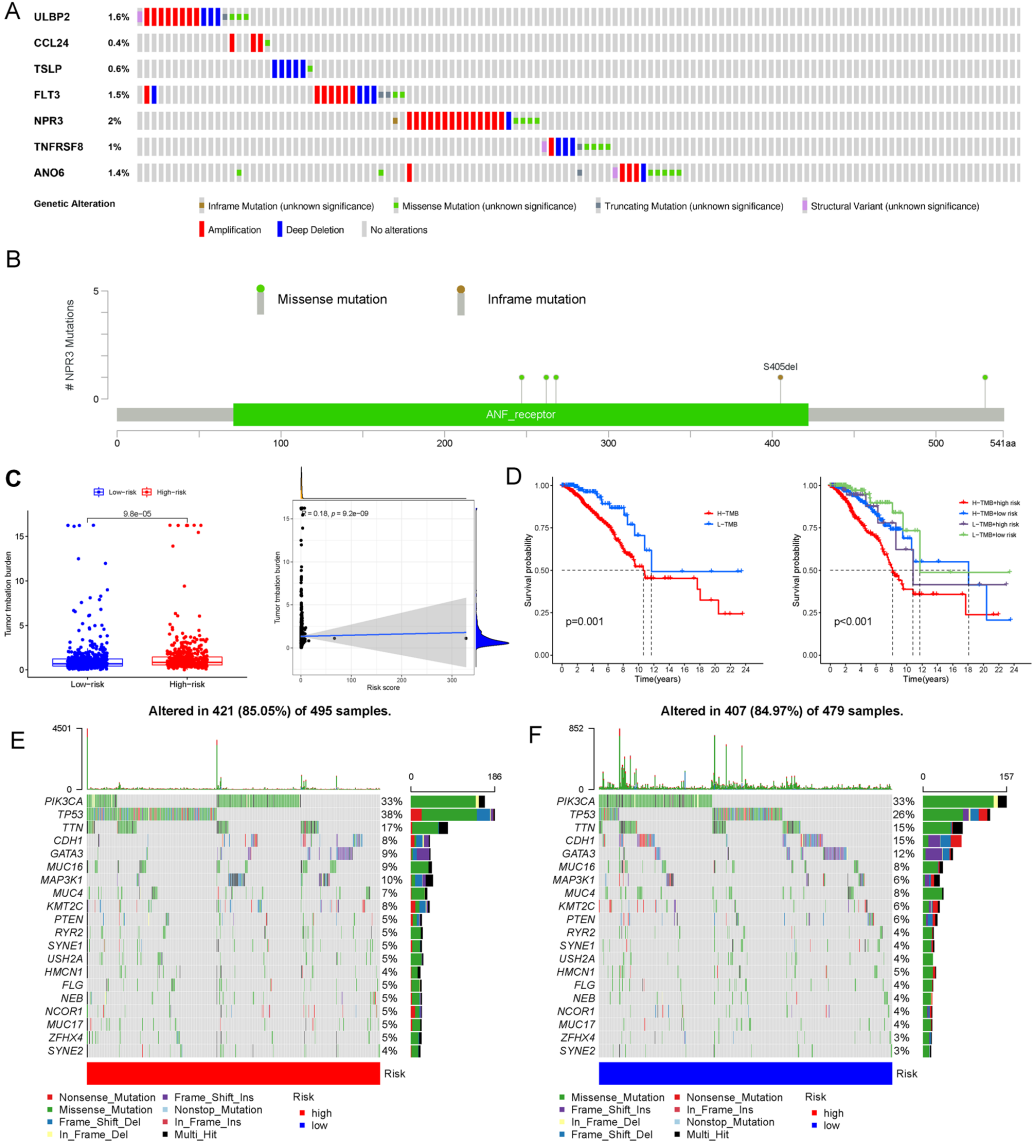
Genetic alteration of immune-related genes (IRGs). In this study, we explored Tumour mutation burden (TMB), somatic mutation, and copy number variations (CNVs) between high- and low-risk groups using the “maftools” R package. Moreover, the cBio Cancer Genomics Portal database (cBioPortal) was performed to assess mutations and CNVs in breast cancer (BC) tissues. (**A**) Analysis of the mutation status of the 7 IRGs in our risk model. (**B**) The most frequent genetic alterations of NPR3 were missense mutation and amplification. (**C**) TMB comparison between the high- and low-risk groups. (**D**) The different survival probabilities for high-risk score BC patients with high TMB and BC patients with both low-risk score and low TMB. (**E**) The mutation frequency and classification of the top 20 gene mutations in the high-risk group. (**F**) The mutation frequency and classification of the top 20 gene mutations in the low-risk group. *IRG* immune-related gene, *TMB* tumour mutation burden, *CNV* copy number variation, *cBioPortal* cBio Cancer Genomics Portal database, *BC* breast cancer.

The mutation frequency of these 7 IRGs was relatively low, and the highest mutation frequency was 2% for NPR3 (Fig. 7A). In addition, the most frequent genetic alteration type was amplification (Fig. 7B). The NPR3 mutations were predicted to be missense mutations and in-frame mutations, and the majority of NPR3 mutations were localized in the ANF receptor (Fig. 7B). BC patients in the high-risk group had a higher tumour mutation burden (TMB) than those in the low-risk group (Fig. 7C). Moreover, as the risk score increased, TMB occurred more frequently in BC patients. Compared with the patients with a high risk score and high TMB, the BC patients with a low risk score and low TMB exhibited a longer survival time and a better prognosis (Fig. 7D). These results validated that both risk score and TMB were tightly associated with the efficacy of ICI treatment. Subsequently, we assessed the mutation frequency and classification and revealed that 421 of 495 (85.05%) BC samples in the high-risk group and 407 of 479 (84.97%) BC samples in the low-risk group had genetic mutations (Fig. 7E,F). In the high-risk group, PIK3CA had a high mutation rate (33%), which was just below that of TP53 (38%) (Fig. 7E). In the low-risk group, PIK3CA had the highest mutation rate (33%) (Fig. 7F). Moreover, the most common genetic alteration type was missense mutation.

### Analysis of TME and tumour immunity between the high- and low-risk groups

**Figure 8:**
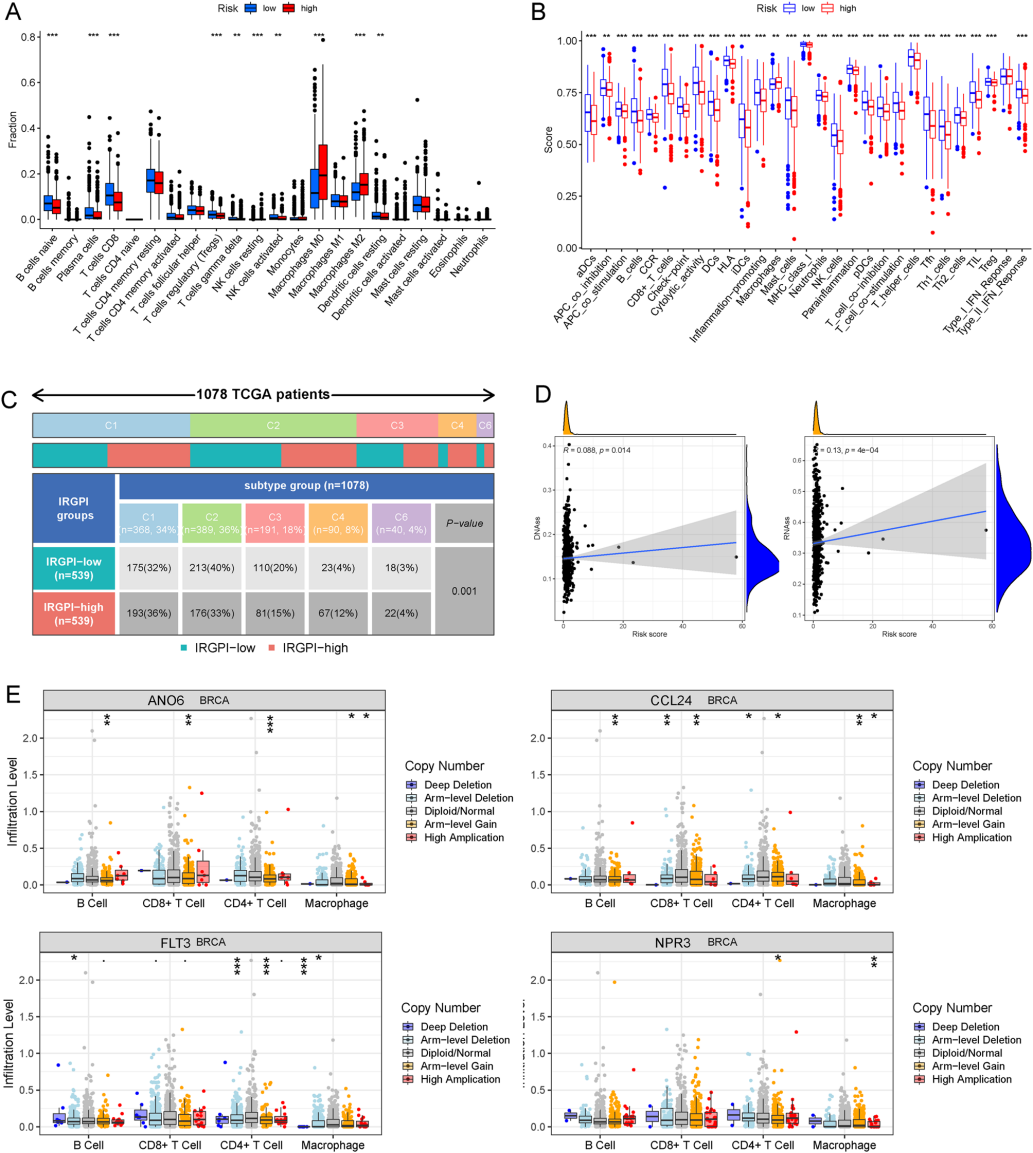
Patients with high- and low-risk scores had different immune statuses. Comparison of the single-sample gene set enrichment analysis (ssGSEA) scores of 22 types of immune cells (**A**) and 13 immune-related pathways (**B**) between low- (blue box) and high-risk (red box) groups in The Cancer Genome Atlas (TCGA) project. (**C**) Immune-related gene prognostic index (IRGPI) differed among subtypes in the high and low-risk groups. (**D**) The tumour stemness of breast cancer (BC) patients between the high- and low-risk groups. (**E**) The relationship between copy number variations (CNVs) of 4 immune-related genes (IRGs) (ANO6, CCL24, FLT3, and NPR3) and the level of infiltration of B cells, T cells, and macrophages in BC. *P < 0.05; **P < 0.01; ***P < 0.001. *ssGSEA* single-sample gene set enrichment analysis, *TCGA* The Cancer Genome Atlas, *IRGPI* immune-related gene prognostic index, *BC* breast cancer, *CNV* copy number variation, *IRG* immune-related gene.

To further clarify the potential relationships between the risk score and TME immune features, we quantified the enrichment scores of 22 immune cell types and 13 immune cell-related functions between the two risk groups by using single-sample gene set enrichment analysis (ssGSEA). BC patients in the high- and low-risk groups showed different infiltration levels of immune cells, including naïve B cells, plasma cells, CD8 + T cells, Tregs, gamma delta T cells, resting natural killer (NK) cells, activated NK cells, M0 macrophages, M2 macrophages and resting dendritic cells (DCs) (Fig. 8A). Intriguingly, among the 22 immune cell types, memory B cells, naïve B cells, plasma cells, follicular helper T cells, CD4 memory resting T cells, M0 macrophages, and M2 macrophages were confirmed to be associated with the OS of BC patients (Figure S2). Moreover, immune cell-related functions were significantly different between the two risk groups (Fig. 8B). Consensus immune subtyping summarized 6 subtypes, including wound healing (C1), IFN-gamma dominant (C2), inflammatory (C3), lymphocyte depleted (C4), immunological quiet (C5), and TGF-beta dominant (C6). Previous studies have confirmed that C3 presents the best prognosis, while C4 presents the least favourable prognosis^14^. The C3 subtype had a higher IRG prognostic index (IRGPI) in low-risk patients, while the C4 subtype had a higher IRGPI in high-risk patients (P  = 0.001, chi-square test; Fig. 8C). In addition, the tumour stemness of BC patients was different between the high- and low-risk groups, as the risk score was significantly positively correlated with the RNA stemness score (RNAss) and DNA stemness score (DNAss) (P < 0.05; Fig. 8D). Then, as somatic copy number alterations (SCNAs) commonly occurred in BC, the SCNA module was used to explore the relationship between the copy number variations (CNVs) of 4 IRGs and infiltration of B cells, T cells, and macrophages in BC patients. The increase in these 4 IRGs in CNVs in different dimensions, including deep deletion, arm-level deletion, arm-level gain, and high amplification was correlated with infiltration of BC immune cells (Fig. 8E), which suggested that these IRGs could act as targets for immunotherapy.

### Analysis of different immunotherapy effects between high- and low-risk groups

**Figure 9:**
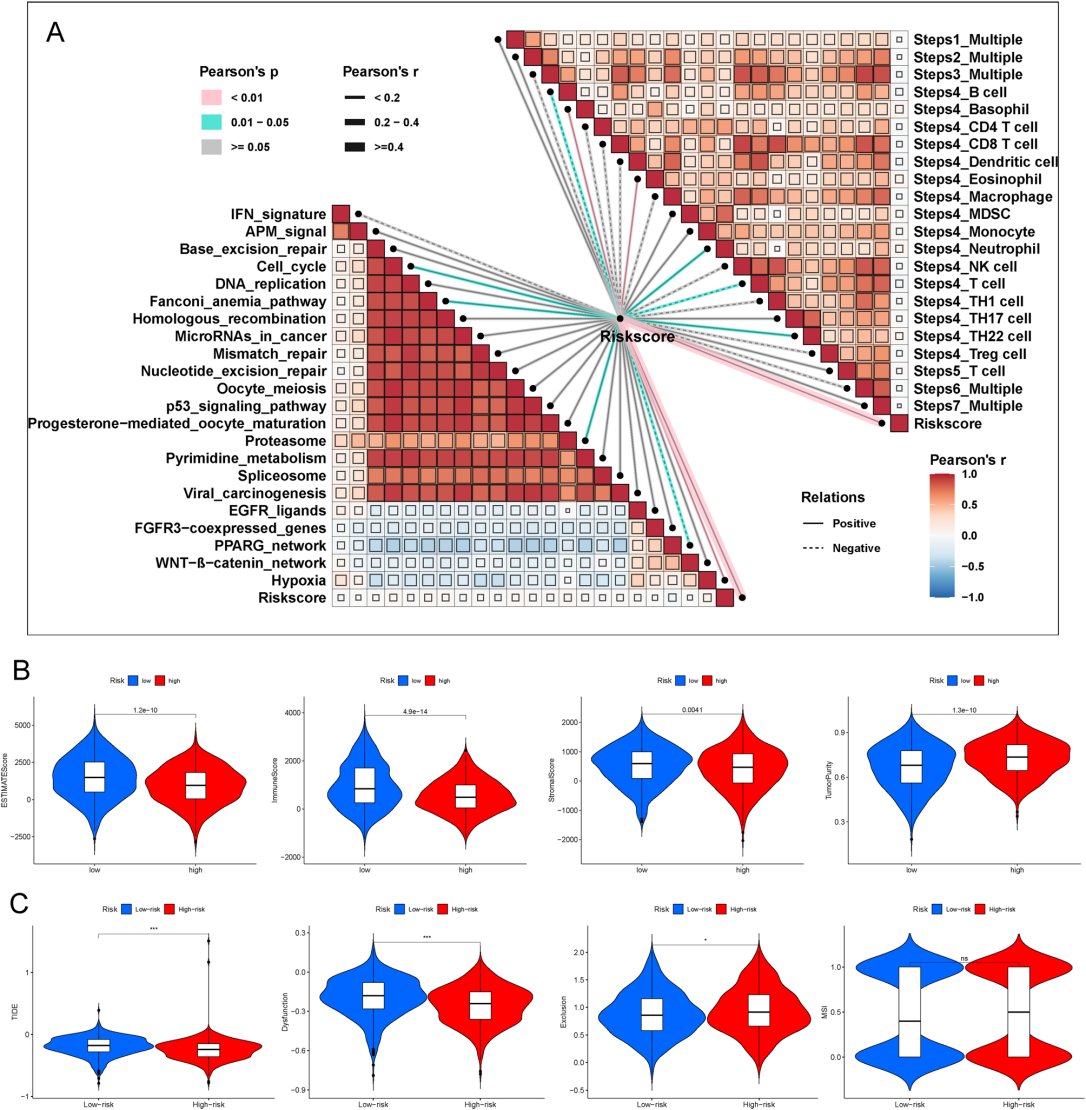
Analysis of immunotherapy effect. (**A**) Correlations between the risk score and enrichment score of the immunotherapy prediction pathway and hallmark gene set. The annotation of genes related cancer-immunity cycle was downloaded from Tracking Tumor Immunephenotype (http://biocc.hrbmu.edu.cn/TIP/index.jsp). (**B**) The immune score, stromal score, tumour purity, and ESTIMATE score were used to quantify the distinct immune statuses between the high- and low-risk groups. (**C**) The Tumour Immune Dysfunction, and Exclusion (TIDE) algorithm was used to predict the immunotherapeutic benefit of The Cancer Genome Atlas (TCGA)-breast cancer (BC) patients. *TIDE* tumour immune dysfunction, and exclusion, *TCGA* The Cancer Genome Atlas, *BC* breast cancer.

First, we calculated enrichment scores for known immunotherapy-related positive signatures and verified that the risk score was positively associated with all of these signatures. The results showed that patients in high-risk groups may be better candidates for antitumour immunotherapy (Fig. 9A). In addition, we analysed the correlation between the risk score and the hallmark gene set score (Fig. 9A). The immune score, stromal score, tumour purity, and ESTIMATE score (stromal score combined with immune score) in BC patients were calculated by the ESTIMATE algorithm to explore the correlation between the two risk groups and tumour immune status. The low-risk group had higher ESTIMATE scores, immune scores, and stromal scores, while the high-risk group had higher tumour purity (P < 0.05) (Fig. 9B). Subsequently, the TIDE algorithm was processed to assess whether the risk score could predict the immunotherapeutic benefit of BC patients. The high-risk group had a higher TIDE prediction score, indicating that the BC patients in the high-risk group were prone to escaping immunological surveillance and could benefit more from ICI therapy (Fig. 9C). Additionally, the low-risk group had a higher T-cell dysfunction score, while the high-risk group had a higher T-cell exclusion score. However, there was no significant difference in microsatellite instability (MSI) between the two risk groups (Fig. 9C).

### Response to immune checkpoint inhibitors in the high- and low-risk groups

**Figure 10:**
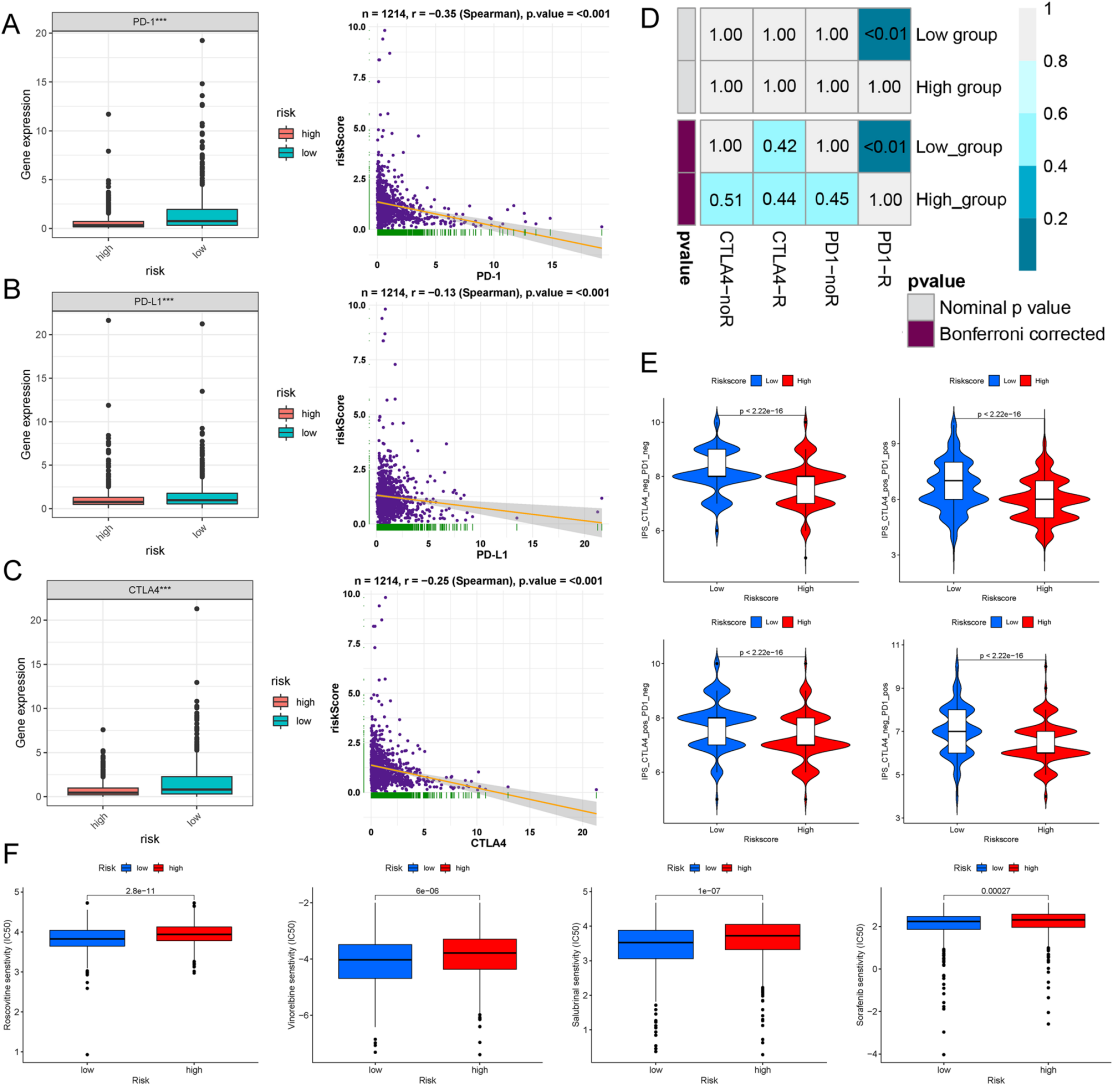
The response to immune checkpoint inhibitors. (**A**–**C**) Relative expression and correlation analysis of three immune checkpoints (PD-1, PD-L1, and CTLA-4) between high- and low-risk groups of breast cancer (BC) patients. (**D**) The response to anti-PD-1 and anti-CTLA-4 immunotherapy in the high- and low-risk groups was evaluated via the submap algorithm. (**E**) The immunophenoscore (IPS) was used to predict patient response to immune checkpoint inhibitors. (**F**) The 7 immune-related genes (IRGs) in our risk model were significantly correlated with the most common chemotherapeutic drugs. *BC* breast cancer, *IPS* immunophenoscore, *IRG* immune-related gene.

First, the expression levels of immune checkpoints (PD-1, PD-L1, and CTLA-4) were detected between the high- and low-risk groups. BC patients with low risk scores showed modestly increased PD-1 (P  < 0.001), PD-L1 (P < 0.001), and CTLA-4 (P  < 0.001) levels. Moreover, the correlation analysis indicated that the risk score had a significant negative correlation with the expression of CTLA-4, PD-1, and PD-L1 (Fig. 10A–C). Then, the submap algorithm was applied to assess the response to anti-PD-1 and anti-CTLA-4 immunotherapy in the high- and low-risk groups. Interestingly, we found that the low-risk group may benefit more from anti-PD-1 treatment (Bonferroni corrected P < 0.01) (Fig. 10D).

The immunophenoscore (IPS) is an evaluation strategy by which machine learning algorithms predict patient response to immune checkpoint inhibitors. Given that there was no definitive information available in the TCGA BC dataset for evaluating ICI treatment, samples were sorted into 4 IPS groups (CTLA-4_neg_PD-1_neg, CTLA-4_pos_PD-1_neg, CTLA-4_neg_PD-1_pos, and CTLA-4_pos_PD-1_pos) to reflect the sensitivity of BC patients to anti-PD-1 and anti-CTLA-4 therapy. In the study, the BC patients in the low-risk group were more sensitive to anti-PD-1 (P  < 2.22^e−16^) and anti-CTLA-4 therapy (P  < 2.22^e−16^), and the same conclusion was reached with combination treatment of anti-PD-1 and anti-CTLA-4 (Fig. 10E). These patients might benefit more from ICI treatment. Interestingly, BC patients in the high-risk group were correlated with a higher IC50 of the chemotherapeutics etoposide and rapamycin (P  < 0.001) (Fig. 10F). As presented in Fig. 10F, there was a significant association of our IRGs with most common chemotherapeutic drugs. These results verified that the proposed risk model in our study could serve as an effective method for predicting the chemotherapeutic sensitivity of BC patients.

### Validation of the predictive ability of the risk model in an external clinical cohort

**Figure 11:**
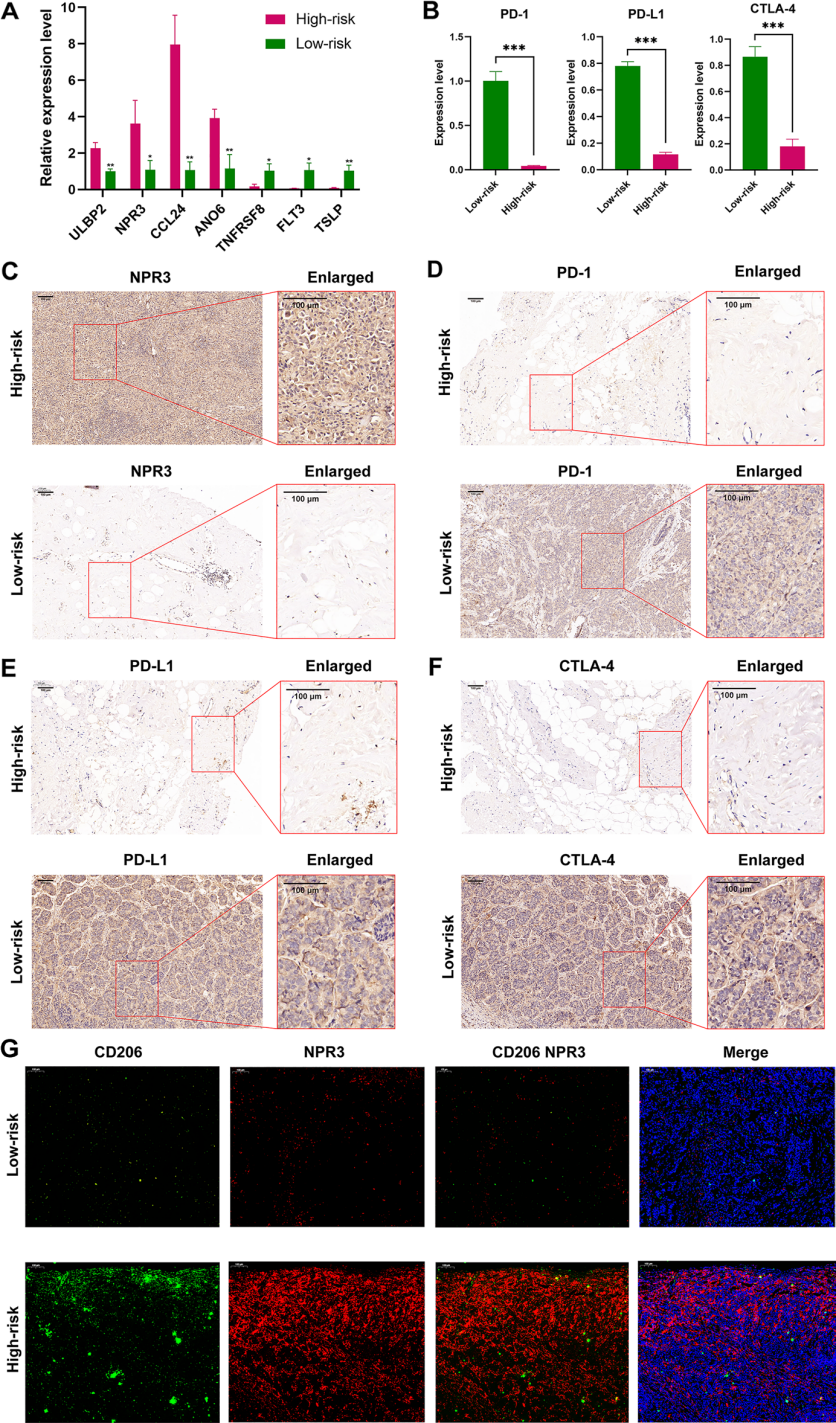
Validation of the predictive ability of the risk model in an external cohort. (**A**) The relative expression of the 7 immune-related genes (IRGs) in breast cancer (BC) samples through quantitative reverse transcription polymerase chain reaction (qRT‒PCR) (n = 30). * indicated P < 0.05, ** indicated P < 0.01, P value based on t-test. (**B**) The expression levels of PD-1, PD-L1, and CTLA-4 were decreased in the high-risk group, as evaluated by qRT‒PCR (n = 30). *** indicated P < 0.001, P value based on t-test. The immunohistochemistry (IHC) results revealed that the expression of NPR3 (**C**) was increased but that of PD-1 (**D**), PD-L1 (**E**), and CTLA-4 (**F**) was decreased in the high-risk group. (**G**) The immunofluorescence (IF) assay demonstrated that M2 macrophages were more abundant in high-risk patients. *IRG* immune-related gene, *BC* breast cancer, *qRT‒PCR* quantitative reverse transcription polymerase chain reaction, *IHC* immunohistochemistry, *IF* immunofluorescence.

A clinical cohort of 30 BC patients with different clinical stages was constructed to verify the predictive ability of the risk score. First, the relative expression levels of the 7 IRGs in our risk model were remarkably different between BC samples in different stages through qRT‒PCR (Fig. 11A). The expression levels of PD-1, PD-L1, and CTLA-4 were detected between the high- and low-risk groups (Fig. 11B). The results of IHC further confirmed that the expression of NPR3 was increased but that of PD-1, PD-L1, and CTLA-4 was decreased in the high-risk group in comparison to the low-risk group (Fig. 11C–F). The IF assay further demonstrated that M2 macrophages were more abundant in high-risk patients, and the CD206 and NPR3 coexpression rate was higher in high-risk patients (Fig. 11G).

### NPR3 promotes the proliferation and migration of BC cells and inhibits their apoptosis

**Figure 12:**
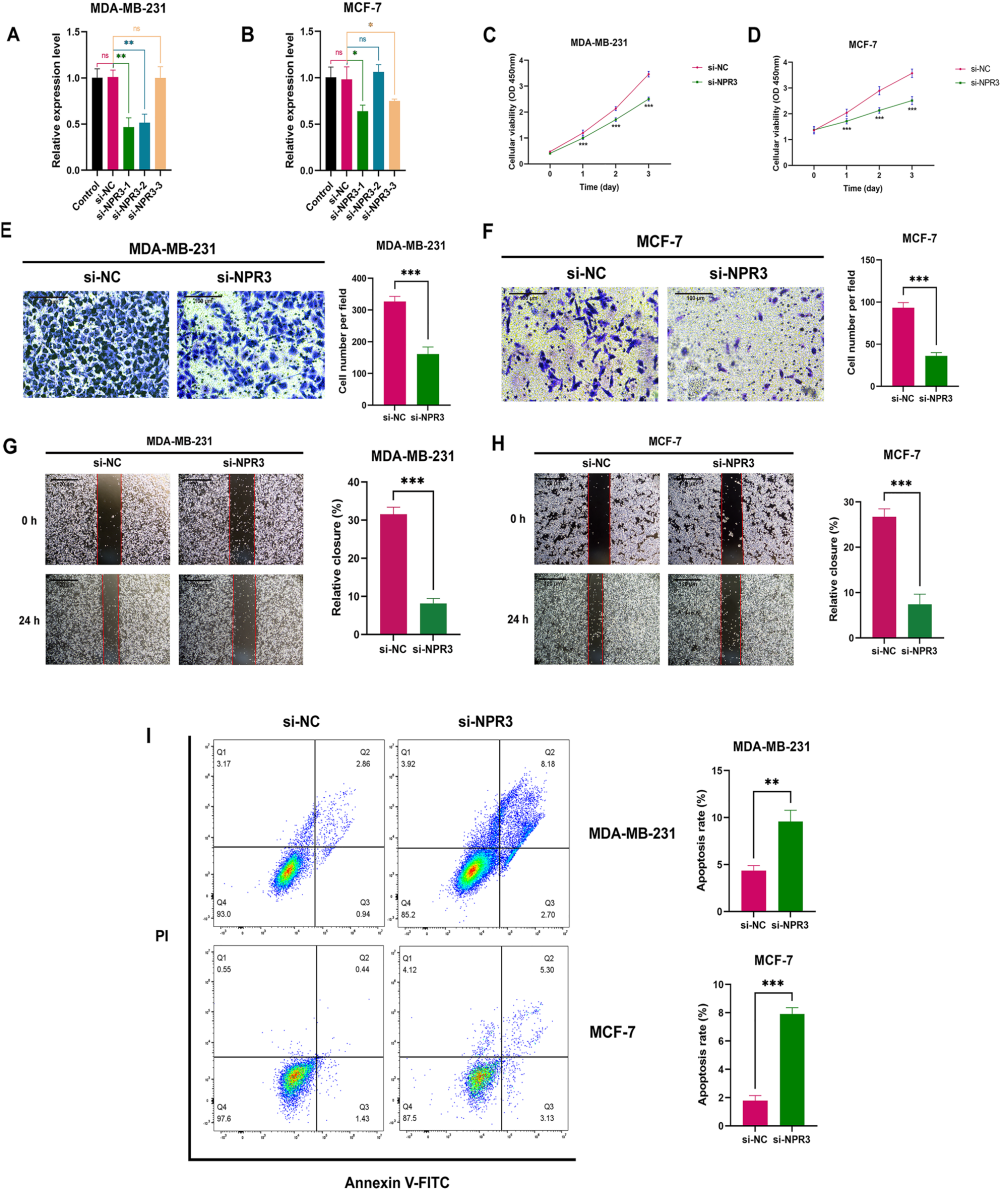
The effect of NPR3 on the proliferation, migration, and apoptosis of breast cancer (BC) cells. The results of quantitative reverse transcription polymerase chain reaction (qRT‒PCR) showed that si-NPR3-1 successfully knocked down the expression of NPR3 in MDA-MB-231 (**A**) and MCF-7 (**B**) cells (n = 3). * indicated P < 0.05, ** indicated P < 0.01, ns indicated no significance, P value based on t-test. The depletion of NPR3 observably suppressed the proliferation ability in both MDA-MB-231 (**C**) and MCF-7 (**D**) cells compared with the si-NC group (n = 3). *** indicated P < 0.001, P value based on t-test. The knockdown of NPR3 significantly decreased the migration of MDA-MB-231 (**E**,**G**) and MCF-7 (**F**, **H**) cells (n = 3). ** indicated P < 0.01, *** indicated P < 0.001, P value based on t-test. (**I**) Flow cytometry indicated that NPR3 deletion could increase apoptosis in MDA-MB-231 and MCF-7 cells (n = 3). ** indicated P < 0.01, *** indicated P < 0.001, P value based on t-test. All experiments were implemented separately in triplicate. *BC* breast cancer, *qRT‒PCR* quantitative reverse transcription polymerase chain reaction.

To further explore the role of NPR3 in BC cells, we examined the effect of the NPR3 gene on BC proliferation, migration, and apoptosis. si-NPR3-1 successfully knocked down the expression of NPR3 in MDA-MB-231 and MCF-7 cells (Fig. 12A,B). CCK-8 analysis revealed that the depletion of NPR3 suppressed the proliferation ability of both MDA-MB-231 and MCF-7 cells compared with the si-NC group (Fig. 12C,D). Moreover, the transwell migration assay and wound healing assay demonstrated that NPR3 knockdown significantly decreased the migration of MDA-MB-231 and MCF-7 BC cells (Fig. 12E–H). The flow cytometry results showed that NPR3 significantly inhibited BC cell apoptosis (Fig. 12I).

## Discussion

Large amounts of evidence have proven that IRGs are differentially expressed in different tumours and participate in the evolution of tumours. IRG-based models are considered to have superb prognostic value for cancers. In the present study, we intended to comprehensively investigate IRGs and their relationship with the tumour immune environment through bioinformatics analysis and validation in BC samples. Specifically, we successfully established a model based on 7 IRGs (ULBP2, CCL24, TSLP, FLT3, NPR3, TNFRSF8, and ANO6), which possessed robust performance for judging BC prognosis. This capability was superior to that of conventional clinical predictors, such as age, survival status, clinical stage, and TNM stage.

These 7 IRGs have distinct expression patterns and molecular functions in cancers. ULBP2 is a newly discovered family of human NKG2D ligands that play an important role in the antitumour immune response^15^. The chemokine CCL24 binds to its only receptor CCR3 and thus participates in atopic diseases, parasitic infections and systemic disease processes^16^. In addition, CCL24 expression is elevated in some tumour tissues and plasma and can be used as a potential therapeutic marker for tumour diagnosis. TSLP belongs to the IL-2 cytokine family of type I cytokines and is important for the initiation and maintenance of Th2-mediated chronic inflammatory responses^17^. FLT3 represents a member of the class III receptor tyrosine kinase family, and somatic mutation and amplification of FLT3 is an important phenomenon associated with tumour development in solid tumours^18^. TNFRSF8, also known as CD30, is expressively related to activated T and B cells and is abnormally expressed in lymphoid malignancies and other solid tumours^19^. The extensive expression of ANO6 provides support for various Ca^2+^-dependent functions^20^. Aberrant expression of ANO6 is involved in tumour metastasis and is closely correlated with ERK signalling activation.

Emerging studies have verified that prognostic models constructed from IRGs have excellent predictive ability. This is probably because the key IRGs not only affect the proliferation, differentiation, metastasis, and stemness of tumour cells themselves but also impact the recruitment, infiltration, proliferation, and killing functional properties of immune cells^21,22^. Then, abnormally expressed IRGs can reflect the changes in the tumour environment as a complicated biological system to a certain extent. For instance, Tan et al. constructed a 9 IRG panel in triple-negative breast cancer (TNBC), and the related nomogram could assess the axillary lymph node (ALN) status preoperatively with better predictive ability than the IRG model alone^23^. Feng et al. revealed that 14 IRGs were correlated with the OS of patients with colorectal cancer (CRC) after chemotherapy, and the constructed model could predict immune status and chemotherapy sensitivity^24^. Consistent with these studies, our risk model was an independent prognostic indicator with higher prognostic efficiency in evaluating OS of BC than other independent clinicopathological variables, including age, clinical stage, and TNM stage. In addition, the risk score could further stratify BC patients in a given clinical or TNM stage group to provide additional information on prognosis.

IRGs are crucial participants in tumour immune microenvironment remodelling and tumour progression. Thus, the IRG-based model can also reflect different immune infiltration statuses and cell function characteristics. Zhu et al. also proposed a risk model based on 12 IRGs, in which risk scores were inversely associated with the infiltration level of B cells, CD4+T cells, CD8+T cells, neutrophils, and DCs^25^. Wang et al. established a model comprising 5 IRGs (CCL25, IL29, TDGF3, GPR44, and GREM2)^26^. This study demonstrated that the risk score was a robust tool in predicting TNBC patient outcomes and could reflect differences in the abundance of common lymphocytes and expression ratios. Zhao et al. presented a risk model based on 27 IRGs that indicates that immune cell infiltration in the TME was markedly decreased in high-risk patients^27^. In our study, different immune pathways and functions were enriched in the high- and low-risk BC groups. In particular, BC patients in the high-risk and low-risk groups showed differences in the levels of immune cell infiltrates, such as naïve B cells, plasma cells, CD8+T cells, Tregs, resting NK cells, and activated NK cells. Moreover, the results suggested that the high-risk group might benefit more from anti-PD-1 treatment. Collectively, these results suggest that these 7 IRGs could act as effective targets for personalized immunotherapy.

NPR3 is a clearance receptor that mediates the degradation of natriuretic peptides while functioning as a tumour suppressor or promoter in specific cancer types^28^. For example, in CRC, the lncRNA BCYRN1 promotes tumour cell proliferation by upregulating NPR3, which confirms that NPR3 is a promoter in CRC^29^. However, Li et al. reported that NPR3 expression was downregulated in osteosarcoma and suppressed osteosarcoma progression by inhibiting the PI3K/AKT pathway and was involved in POU2F1 regulation^30^. NPR3 was also reported to exert a tumour-suppressive effect by regulating MRCCAT1-mediated clear cell renal cell carcinoma metastasis^31^. Here, our in vitro experiments showed that NPR3 could promote the proliferation and migration of TNBC cells and ER+ cells. This result emphasized that NPR3 was a facilitator in BC progression. This result emphasized that NPR3 was a facilitator of BC progression. This phenomenon may be partially due to the fact that breast tumours are a highly heterogeneous entity with a high frequency of genetic mutations; genes related to NPR3 are upregulated in the BC TME and downregulated in other tumours. Intriguingly, many risk models include NPR3 as a key indicator. Xu et al. also established an NPR3-containing 7-IRG risk model, and the risk score presented excellent predictive ability in gastric cancer and revealed the status of the tumour immune microenvironment^32^. Similarly, Wang et al. proposed a model consisting of 4 stemness-related genes (PSMB9, CXCL13, NPR3, and CDKN2C) to identify targets of BC stem cells and improved the therapeutic effect^33^. In our study, NPR3 with the other 6 IRGs constituted an excellent model with excellent prognostic and TME-predictive value for BC.

However, this study has some limitations that need to be addressed. First, we used a variety of bioinformatics approaches to verify the efficacy and clinical value of this model. Although this study adopted a small number of BC samples for verification, it only preliminarily confirmed the reliability at the clinical level. The practical guiding value for clinical practice is not that thorough. Second, the above 7 IRGs are involved in various aspects of molecular mechanisms, such as immune regulation and ICI treatment, but this study is only a preliminary verification, and its detailed mechanism needs further exploration. Finally, there might be some unknown factors that are difficult to extrapolate based on public database analysis. Further comprehensive investigation in large multicentre cohorts with more external experiments will be facilitate in-depth confirmation of this model.

## Conclusion

In this study, we successfully established a prognostic risk model for BC based on 7 IRGs, possessing the ability to predict BC prognosis and immunotherapy response. Our study provides a reliable integrated model with multiple genes for predicting BC prognosis and might offer novel guidance for the application of immunotherapy and clinical outcomes in BC patients.

## Supplementary Information

Supplementary Figure 1.

Supplementary Figure 2.

Supplementary Legends.

Supplementary Table 1.

Supplementary Table 2.

Supplementary Table 3.

Supplementary Table 4.

## Data Availability

The datasets provided for this study can be found in online repositories. The name and accession number(s) of the repository/repositories can be found in the article/Supplementary Material.

## Abbreviations

BC: Breast cancer
IRG: Immune-related gene
TIDE: Tumour immune dysfunction and exclusion
TCGA: The Cancer Genome Atlas
TNM: Tumour-node-metastasis
TME: Tumour microenvironment
ICI: Immune checkpoint inhibitor
PTC: Papillary thyroid cancer
PFS: Progression-free survival
LASSO: Least absolute shrinkage and selection operator
OS: Overall survival
RNA-Seq: RNA sequencing
PFI: Progression-free interval
DSS: Disease-specific survival
GO: Gene Ontology
KEGG: Kyoto Encyclopedia of Genes and Genomes
GSEA: Gene set enrichment analysis
PCA: Principal component analysis
ROC: Receiver operating characteristic
AUC: Area under the curve
GDSC: Genomics of drug sensitivity in cancer
ATCC: American type culture collection
DMEM: Dulbecco’s modified Eagle medium
FBS: Fetal bovine serum
siRNA: Small interfering RNA
IHC: Immunohistochemistry
HRP: Horseradish peroxidase
IF: Immunofluorescence
DFS: Disease-free survival
WGCNA: Weighted gene co-expression network analysis
MM: Module membership
GS: Gene significance
TMB: Tumour mutation burden
DC: Dendritic cell
IRGPI: Immune-related gene prognostic index
RNAss: RNA stemness score
DNAss: DNA stemness score
SCNA: Somatic copy number alteration
CNV: Copy number variation
MSI: Microsatellite instability
IPS: Immunophenoscore
ALN: Axillary lymph node
CRC: Colorectal cancer
NK: Natural killer
qRT‒PCR: Quantitative reverse transcription polymerase chain reaction
CCK-8: Cell counting kit-8
SD: Standard deviation
FITC: Fluorescein isothiocyanate
PI: Propidium iodide
EDTA: Ethylene diamine-tetra acetic acid
PBS: Phosphate buffer saline
DAB: Diaminobenzidine
DAPI: 4,6-Diamidino-2-phenylindole
HR: Hazard ratio
CI: Confidence interval
TNBC: Triple negative breast cancer
ssGSEA: Single-sample gene set enrichment analysis
cBioPortal: yyCBio Cancer Genomics Portal database

## References

[1] Siegel RL, Miller KD, Jemal A (2020). Cancer statistics, 2020. CA. Cancer J. Clin..

[2] Fisusi FA, Akala EO (2019). Drug combinations in breast cancer therapy. Pharm. Nanotechnol..

[3] Wilson BE, Gorrini C, Cescon DW (2022). Breast cancer immune microenvironment: From pre-clinical models to clinical therapies. Breast Cancer Res. Treat..

[4] Deepak KGK (2020). Tumor microenvironment: Challenges and opportunities in targeting metastasis of triple negative breast cancer. Pharmacol. Res..

[5] Crunkhorn S (2022). Blocking breast cancer metastasis. Nat. Rev. Drug Discov..

[6] Sun J (2021). Characterization of immune landscape in papillary thyroid cancer reveals distinct tumor immunogenicity and implications for immunotherapy. Oncoimmunology.

[7] Majidpoor J, Mortezaee K (2021). The efficacy of PD-1/PD-L1 blockade in cold cancers and future perspectives. Clin. Immunol..

[8] Boman C (2021). Discordance of PD-L1 status between primary and metastatic breast cancer: A systematic review and meta-analysis. Cancer Treat. Rev..

[9] Jin K (2021). Development of prognostic signature based on immune-related genes in muscle-invasive bladder cancer: Bioinformatics analysis of TCGA database. Aging (Albany, NY.).

[10] Repo H (2020). A prognostic model based on cell-cycle control predicts outcome of breast cancer patients. BMC Cancer.

[11] Kanehisa M, Sato Y, Kawashima M, Furumichi M, Tanabe M (2016). KEGG as a reference resource for gene and protein annotation. Nucleic Acids Res..

[12] Kanehisa M, Goto S (2000). KEGG: Kyoto Encyclopedia of Genes and Genomes. Nucleic Acids Res..

[13] Dorward HS (2016). Pharmacological blockade of aquaporin-1 water channel by AqB013 restricts migration and invasiveness of colon cancer cells and prevents endothelial tube formation in vitro. J. Exp. Clin. Cancer Res..

[14] Thorsson V (2018). The immune landscape of cancer. Immunity.

[15] Zhou Y, An H, Wu G (2020). Microrna-6071 suppresses glioblastoma progression through the inhibition of pi3k/akt/ mtor pathway by binding to ulbp2. Oncol. Targets Ther..

[16] Lim SJ (2021). CCL24 signaling in the tumor microenvironment. Adv. Exp. Med. Biol..

[17] Wang WR (2021). Associations among phthalate exposure, DNA methylation of TSLP, and childhood allergy. Clin. Epigenet..

[18] Hasegawa H (2021). FMS-like tyrosine kinase 3 (FLT3) amplification in patients with metastatic colorectal cancer. Cancer Sci..

[19] Cheng J, Zhu H, Choi JK (2017). CD30 expression in pediatric neoplasms, study of 585 cases. Pediatr. Dev. Pathol..

[20] Xuan ZB, Wang YJ, Xie J (2019). ANO6 promotes cell proliferation and invasion in glioma through regulating the ERK signaling pathway. Oncol. Targets Ther..

[21] Dou M (2022). Immune-related genes for predicting future kidney graft loss: A study based on GEO database. Front. Immunol..

[22] Cao L (2022). Exploring immune-related prognostic signatures in the tumor microenvironment of colon cancer. Front. Genet..

[23] Tan W (2020). Construction of an immune-related genes nomogram for the preoperative prediction of axillary lymph node metastasis in triple-negative breast cancer. Artif. Cells Nanomed. Biotechnol..

[24] Feng W (2022). A prognostic model using immune-related genes for colorectal cancer. Front. Cell Dev. Biol..

[25] Zhu T (2020). Construction and validation of an immunity-related prognostic signature for breast cancer. Aging (Albany, NY.).

[26] Wang P (2020). Nomogram personalizes and visualizes the overall survival of patients with triple-negative breast cancer based on the immune genome. Biomed. Res. Int..

[27] Zhao Y, Pu C, Liu Z (2020). Exploration the significance of a novel immune-related gene signature in prognosis and immune microenvironment of breast cancer. Front. Oncol..

[28] Liu L (2016). Salicylic acid receptors activate jasmonic acid signalling through a non-canonical pathway to promote effector-triggered immunity. Nat. Commun..

[29] Gu L, Lu L, Zhou D, Liu Z (2018). Long noncoding RNA BCYRN1 promotes the proliferation of colorectal cancer cells via up-regulating NPR3 expression. Cell. Physiol. Biochem..

[30] Li S (2021). NPR3, transcriptionally regulated by POU2F1, inhibits osteosarcoma cell growth through blocking the PI3K/AKT pathway. Cell. Signal..

[31] Li JK (2017). Long noncoding RNA MRCCAT1 promotes metastasis of clear cell renal cell carcinoma via inhibiting NPR3 and activating p38-MAPK signaling. Mol. Cancer.

[32] Xu X (2021). A signature of seven immune-related genes predicts overall survival in male gastric cancer patients. Cancer Cell Int..

[33] Wang WJ (2020). Assessing the prognostic value of stemness-related genes in breast cancer patients. Sci. Rep..

