# Supplementary figures and images for: A novel immune score model predicting the prognosis and immunotherapy response of breast cancer
Source: Sci Rep. 2023 Apr 19;13:6403. doi: 10.1038/s41598-023-31153-2 (PMC10115816; doi:10.1038/s41598-023-31153-2)

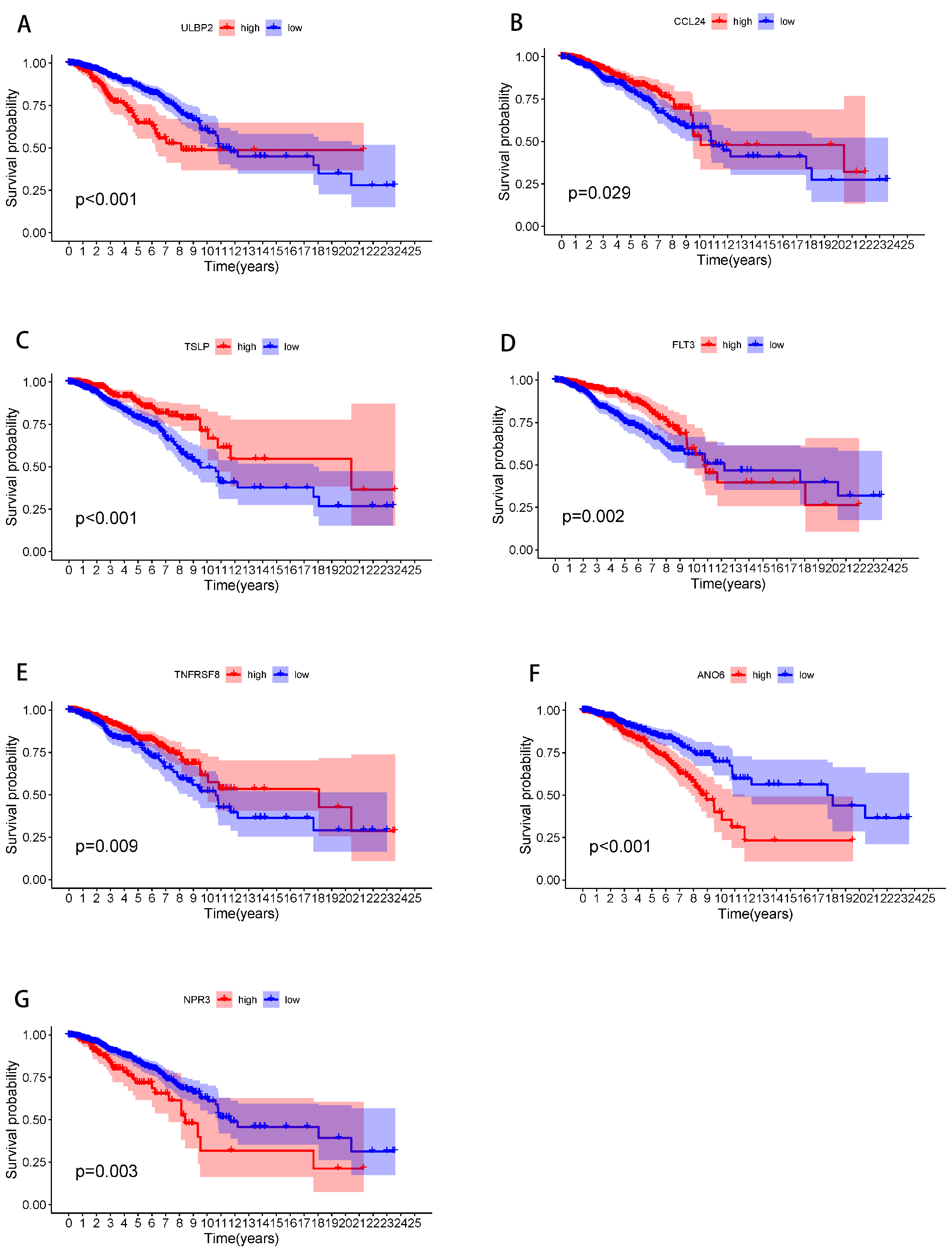

Supplement: Supplementary file 1 — Supplementary Figure 1. [file 41598_2023_31153_MOESM1_ESM.tif]

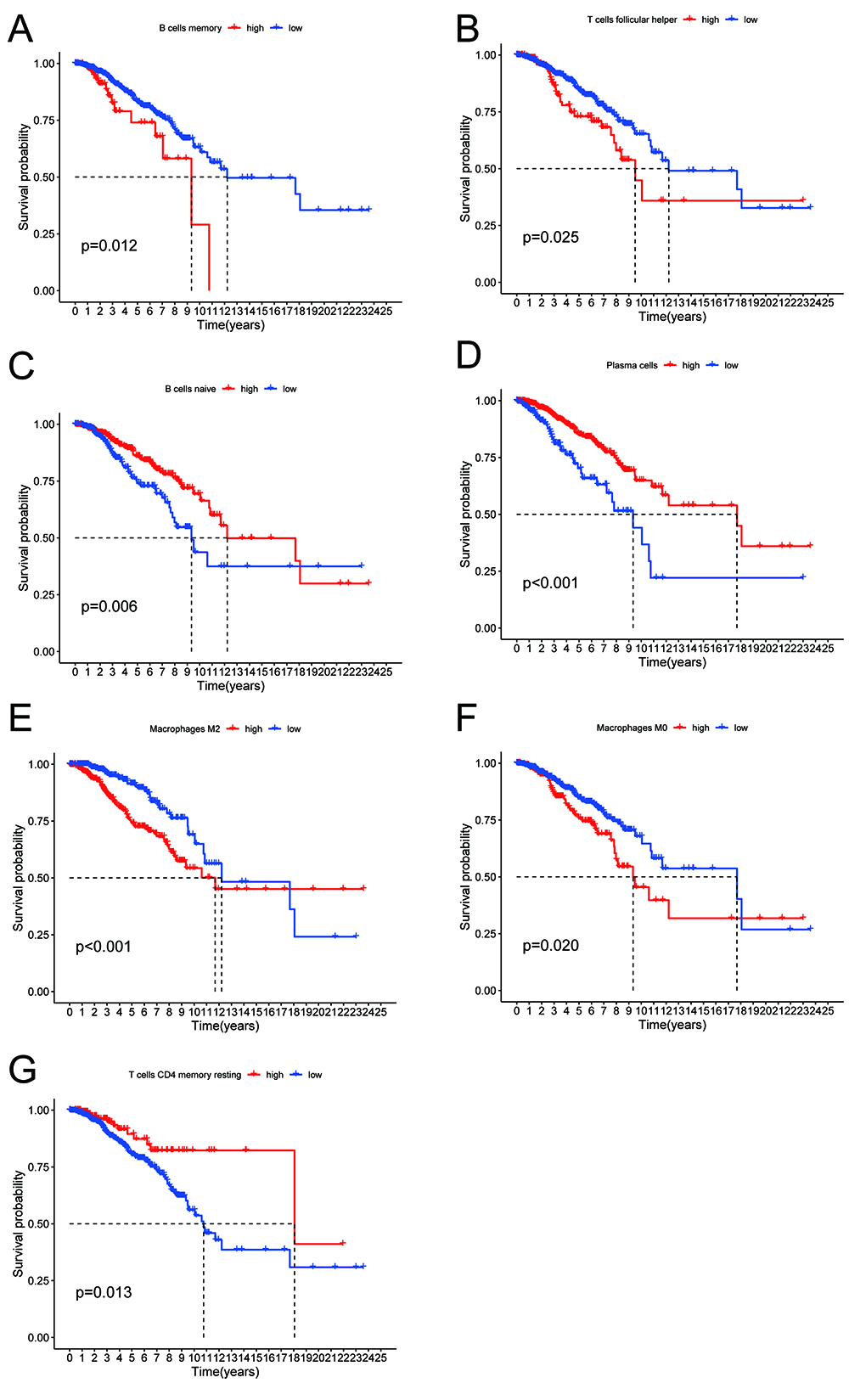

Supplement: Supplementary file 2 — Supplementary Figure 2. [file 41598_2023_31153_MOESM2_ESM.tif]
