# Supplementary material for: A novel immune score model predicting the prognosis and immunotherapy response of breast cancer
Source: Sci Rep. 2023 Apr 19;13:6403. doi: 10.1038/s41598-023-31153-2 (PMC10115816; doi:10.1038/s41598-023-31153-2)
Supplement: Supplementary file 3 — Supplementary Legends. [file 41598_2023_31153_MOESM3_ESM.docx]

**Figure S1. The Kaplan-Meier survival analysis of breast cancer (BC) patients with the different expression levels of 7 immune-related genes (IRGs).** (A) ULBP2. (B) CCL24. (C) TSLP. (D) FLT3. (E) TNFRSF8. (F) ANO6. (G) NPR3. **Abbreviations:** BC, Breast cancer; IRG, Immune-related gene.

**Figure S2. Relationship between the immune cells and survival and prognosis in breast cancer (BC) patients.** (A) B cells memory. (B) T cells follicular helper. (C) B cells naive . (D) Plasma cells. (E) Macrophages M2. (F) Macrophages M0. (G) T cells CD4 memory resting. **Abbreviations:** BC, Breast cancer.
