# Supplementary material for: A novel immune score model predicting the prognosis and immunotherapy response of breast cancer
Source: Sci Rep. 2023 Apr 19;13:6403. doi: 10.1038/s41598-023-31153-2 (PMC10115816; doi:10.1038/s41598-023-31153-2)
Supplement: Supplementary file 6 — Supplementary Table 3. [file 41598_2023_31153_MOESM6_ESM.docx]

**Table S3.** siRNA sequences for NPR3.

| Gene | sense 5’-3’ | antisense 5’-3’ |
| --- | --- | --- |
| Si-NC | CAGAAGAATGGTACAAATCCAAG | CTTCGTTCAGTATGTTAATCGT |
| si-NPR3 | GAGAAACAAGGGCTCAATA | TATTGAGCCCTTGTTTCTC |
| si-NPR3 | CGTCTTGGCTCTACATGAA | TTCATGTAGAGCCAAGACG |
| si-NPR3 | CTAGGAGCTGGCTTGCTAA | TTAGCAAGCCAGCTCCTAG |
