# Supplementary material for: A novel immune score model predicting the prognosis and immunotherapy response of breast cancer
Source: Sci Rep. 2023 Apr 19;13:6403. doi: 10.1038/s41598-023-31153-2 (PMC10115816; doi:10.1038/s41598-023-31153-2)
Supplement: Supplementary file 7 — Supplementary Table 4. [file 41598_2023_31153_MOESM7_ESM.docx]

**Table S4.** Primers for qRT-PCR.

| Gene | Forward | Reverse |
| --- | --- | --- |
| NPR3 | AGACTACGCCTTCTTCAACATTG | GCTTCAAAGTCGTGTTTGTCTCC |
| ULBP2 | GTGGTGGACATACTTACAGAGC | CTGCCCATCGAAACTGAACTG |
| CCL24 | ACATCATCCCTACGGGCTCT | CTTGGGGTCGCCACAGAAC |
| ANO6 | AAATTGCCTCTGAAACCCAATGA | GCTTTCGTCTACACTGAGGACTT |
| TNFRSF8 | TCCACGGAGCACACCAATAAC | ACTGAGAGCATGACATCGCTG |
| FLT3 | AGGGACAGTGTACGAAGCTG | GCTGTGCTTAAAGACCCAGAG |
| TSLP | ATGTTCGCCATGAAAACTAAGGC | GCGACGCCACAATCCTTGTA |
| PD-1 | CCAAGGCGCAGATCAAAGAGA | AGGACCCAGACTAGCAGCA |
| PD-L1 | TGGCATTTGCTGAACGCATTT | TGCAGCCAGGTCTAATTGTTTT |
| CTLA-4 | GCCCTGCACTCTCCTGTTTTT | GGTTGCCGCACAGACTTCA |
| GAPDH | GGAGCGAGATCCCTCCAAAAT | GGCTGTTGTCATACTTCTCATGG |
